# Proteomic analysis of near‐isogenic lines reveals key biomarkers on wheat chromosome 4B conferring drought tolerance

**DOI:** 10.1002/tpg2.20343

**Published:** 2023-05-18

**Authors:** Sina Nouraei, Md Sultan Mia, Hui Liu, Neil C. Turner, Javed M. Khan, Guijun Yan

**Affiliations:** ^1^ UWA School of Agriculture and Environment The University of Western Australia Crawley Western Australia Australia; ^2^ The UWA Institute of Agriculture The University of Western Australia Crawley Western Australia Australia; ^3^ Department of Primary Industries and Regional Development South Perth Western Australia Australia; ^4^ Proteomics International Crawley Western Australia Australia; ^5^ Harry Perkins Institute of Medical Research QEII Medical Centre, The University of Western Australia Crawley Western Australia Australia

## Abstract

Drought is a major constraint for wheat production that is receiving increased attention due to global climate change. This study conducted isobaric tags for relative and absolute quantitation proteomic analysis on near‐isogenic lines to shed light on the underlying mechanism of *qDSI.4B.1* quantitative trait loci (QTL) on the short arm of chromosome 4B conferring drought tolerance in wheat. Comparing tolerant with susceptible isolines, 41 differentially expressed proteins were identified to be responsible for drought tolerance with a *p*‐value of < 0.05 and fold change >1.3 or <0.7. These proteins were mainly enriched in hydrogen peroxide metabolic activity, reactive oxygen species metabolic activity, photosynthetic activity, intracellular protein transport, cellular macromolecule localization, and response to oxidative stress. Prediction of protein interactions and pathways analysis revealed the interaction between transcription, translation, protein export, photosynthesis, and carbohydrate metabolism as the most important pathways responsible for drought tolerance. The five proteins, including 30S ribosomal protein S15, SRP54 domain‐containing protein, auxin‐repressed protein, serine hydroxymethyltransferase, and an uncharacterized protein with encoding genes on 4BS, were suggested as candidate proteins responsible for drought tolerance in *qDSI.4B.1* QTL. The gene coding SRP54 protein was also one of the differentially expressed genes in our previous transcriptomic study.

## Core Ideas

- Near‐isogenic lines of wheat for *qDSI.4B.1* QTL conferring drought tolerance were subjected to drought stress.
- Forty‐one proteins were identified to be responsible for drought tolerance by iTRAQ analysis of opposite isolines.
- Transcription, translation, protein export, photosynthesis, and carbohydrate metabolism were important pathways.
- Five proteins had encoding genes located in the QTL with one of them reported in our previous transcriptomic study.

**Abbreviations**
3PG: 3‐phosphoglycerate
ABA: abscisic acid
ARP: auxin‐repressed protein
C: control
CS: Chinese Spring
D: drought stress
DEPs: differentially expressed proteins
DRP1C: dynamin‐related protein 1C
DS: drought stress
ENPP: ectonucleotide pyrophosphatase/phosphodiesterase family member 1/3
FC: fold change
FDR: false discovery rate
GO: gene ontology
GS: glutamine synthetize
HAO: (S)‐2‐hydroxy‐acid oxidase
iTRAQ: isobaric tags for relative and absolute quantitation
IWGSC: International Wheat Genome Sequencing Consortium
KEGG: Kyoto Encyclopedia of Genes and Genomes
LC: liquid chromatography
LON: ATP‐dependent protease La
MQTL: meta‐quantitative trait loci
MS: mass spectrometry
NILs: near‐isogenic lines
PC: pot capacity
PGMi: phosphoglycerate mutase, co‐factor independent
PPI: protein–protein interaction
PSI: photosystem I
PSPEP: proteomics system performance evaluation pipeline
qRT‐PCR: real‐time polymerase chain reaction
QTL: quantitative trait loci
Rht1: reduced height‐1
RNP: ribonucleoprotein
ROS: reactive oxygen species
RP: ribosomal proteins
RPS15: 30S ribosomal protein S15
RRM: RNA recognition motif
RUBISCO: ribulose‐1,5‐bisphosphate carboxylase/oxygenase
RWC: relative water content
S: susceptible
SHMT: serine hydroxymethyltransferase
SNPs: single nucleotide polymorphisms
SRP54: 54‐kDa protein subunit of the signal recognition particle
SuSy: sucrose synthase
T: tolerant
UDP‐glucose: uridine diphosphate glucose
WW: well‐watered

## 1 INTRODUCTION

Wheat (*Triticum aestivum* L.) with an annual world production of 760 million tonnes (2019) is an important source of calories, fiber, and protein in human diets around the world (FAO, 2021). Drought is among the most important environmental factors that limit wheat growth and production worldwide (Ahmad et al., 2018). Many terrestrial areas are experiencing increased drought stress (DS) as a result of climate change (Seleiman et al., 2021). In addition, the exponential increase in world population puts strong pressure on the agriculture sector to ensure food security and economic development by sufficient food supply (Kulkarni et al., 2017).

The impact of DS on wheat can vary according to drought severity, duration, and growth stage and is very critical during the flowering and grain‐filling phases (terminal drought) (Farooq et al., 2014). Molecular dissection of the complex trait of drought tolerance is vital for improving wheat adaptation to stress conditions (Li et al., 2019). Drought stress can disturb the relationship between sink and source in plant organs and intensively change the morphological, physiological, and biochemical mechanisms (Wang et al., 2016). In response to DS, plants show highly dynamic processes at the levels of genes, proteins, and metabolites, including gene regulation, reactive oxygen species (ROS) scavenging, osmolyte synthesis, cell structure modulation, and hormone induction (Michaletti et al., 2018).

Proteins play an essential role in the plant's phenotype by direct involvement in cell structure and metabolism (Kosová et al., 2018). Because proteins are involved in plant stress responses, proteomic studies can lead to an understanding of the possible relationships between protein changes and plant stress tolerance (S. Wu et al., 2017). Near‐isogenic lines (NILs) are lines with identical genetic backgrounds except for a few specific locations that are used to minimize genetic background effects such as epistatic interactions (Wang et al., 2021). Thus, the comparison of the proteome in drought‐tolerant and susceptible isolines can be used in understanding the underlying mechanism of the targeted locus responsible for drought tolerance (Michaletti et al., 2018). Proteomics is a powerful omics technique that can provide insight into the complex biological mechanisms through analyzing the expression profile of the proteome (whole set of proteins). This technique, coupled with in silico analysis, can be used for protein identification, localization, modification, and protein–protein interactions (PPI) (S. Chen et al., 2018). Isobaric tags for relative and absolute quantitation (iTRAQ) is a powerful high‐throughput technique for quantitative proteome analysis with higher identification rate and reproducibility in comparison to traditional two‐dimensional gel electrophoresis analysis (Gan et al., 2007; Karp et al., 2010). The iTRAQ method has been widely used to study quantitative proteomics of wheat in response to salt (Jiang et al., 2017), heat (Y. Zhang et al., 2018), cold (N. Zhang et al., 2018), and DS (Wang et al., 2019).

Drought tolerance and related traits are complex and polygenic with large genotype‐by‐environment interactions (Verma et al., 2020). A large number of quantitative trait loci (QTLs) have been reported in wheat for grain yield and yield components under water stress through QTL interval mapping and genome‐wide association studies (Czyczyło‐Mysza et al., 2011; Fleury et al., 2010; Gupta et al., 2017; Xu et al., 2017; Yang et al., 2007). However, only some of the QTLs had major effects (explaining ≥20% of the phenotypic variation) and stability (detected in several environments), whereas most were unstable and had only a small effect (Bennett et al., 2012; Gahlaut et al., 2017; Rabbi et al., 2021). In a 2‐year study, a consistent major genomic region *qDSI.4B.1* for yield and yield components under drought stress was reported on the short arm of chromosome 4B (4BS) with a positive allele from wheat cultivar C306 (RGN/CSK3//2*C591/3/C217/N14//C281). This region was responsible for up to 22% of the phenotypic variation in grain yield, shoot biomass, root biomass, and harvest index under DS (Kadam et al., 2012). The *reduced height‐1 (Rht1)* gene associated with the Green Revolution through the reduction of plant height and enhancement of yield is also located on the 4BS chromosome arm (Pearce et al., 2011). Additionally, a previous meta‐QTL (MQTL) study discovered three major locations of MQTL4B.1, MQTL4B.2, and MQTL4B.3 for yield and yield components under various conditions on 4BS, making this location very interesting for further scrutiny (Liu et al., 2020). Mia et al. (2019) employed heterogeneous inbred family analysis coupled with an immature embryo culture–based fast generation technique to develop NILs from a cross between C306 and Dharwar Dry. In a previous study, we discovered six candidate genes at the *qDSI.4B.1* locus by transcriptomic (RNA‐seq) analysis of grains from two pairs of NILs under control and DS conditions (Nouraei et al., 2022). However, because post‐transcriptional modification plays a crucial role in regulating gene expression, the transcriptome examination alone is not sufficient to decipher the complex trait of drought tolerance (Pan et al., 2018). Therefore, the complementary investigation through proteome analysis is necessary to better understand the molecular basis of drought tolerance mediated by this locus.

In this study, we investigated the comparative proteomics of a pair of NILs for *qDSI.4B.1* QTL under control and drought conditions using the iTRAQ technique. The goals of this study were to (i) find candidate proteins responsible for drought tolerance within the *qDSI.4B.1* locus, (ii) determine the expression pattern and molecular function of the candidate proteins, (iii) subsequently integrate the proteomic data into a network of regulatory pathways to systematically illustrate the processes involved in drought tolerance, and (iv) assess the role of post‐transcriptional modification by investigating the consistency between gene expression and protein abundance to provide further insights into the role of the *qDSI.4B.1* QTL conferring drought tolerance in wheat.

## 2 MATERIALS AND METHODS

### 2.1 Plant materials, growth conditions, and sampling

Four NIL pairs of wheat (*T. aestivum* L.) developed for an important and consistent QTL of *qDSI.4B.1* on the short arm of chromosome 4B that confers drought tolerance (Mia et al., 2019) were grown. To avoid the effect of a closely linked gene, *Rht‐B1*, controlling plant height, a pair of NILs, qDSI.4B.1‐8, having no significant height differences between tolerant and susceptible isolines were selected. Plants were grown from July to October 2020 in a glasshouse facility of The University of Western Australia in Perth, Western Australia (31°59′ S, 115°49′ E, and 31.5 m above sea level). The experimental design had a completely randomized block structure with three biological replications. Seeds were sown in cylindrical columns containing 1.3 kg air‐dried potting mix (5:2:3 fine composted pine bark:cocopeat:brown river sand, pH ∼ 6.0). Two water treatments, including well‐watered (WW) and DS, were applied: In the WW treatment, pots were kept between 80% and 100% of pot capacity (PC, Passioura, 2006) from sowing to physiological maturity by watering at least every 2 days. In the DS treatment, pots were kept between 80% and 100% of PC from sowing to anthesis when the DS treatment was applied by stopping irrigation for 7 days. The main stem flag leaf was collected at 7 days after anthesis from the WW (control) and DS treatments and stored at −80°C for protein extraction. The average soil water contents at sampling were 89% and 42% of PC in the WW and DS treatments, respectively. The soil water content was similar for tolerant and susceptible isolines in both treatments. DS‐treated pots were re‐irrigated after sampling and kept at 80%–100% of PC until physiological maturity.

**Core Ideas**

- Near‐isogenic lines of wheat for *qDSI.4B.1* QTL conferring drought tolerance were subjected to drought stress.
- Forty‐one proteins were identified to be responsible for drought tolerance by iTRAQ analysis of opposite isolines.
- Transcription, translation, protein export, photosynthesis, and carbohydrate metabolism were important pathways.
- Five proteins had encoding genes located in the QTL with one of them reported in our previous transcriptomic study.

### 2.2 Morphological and physiological measurements

Grain yield, 1000‐kernel weight, and dry weight of aerial parts were measured at physiological maturity and reported in grams per plant. For yield, in each pot, kernels on all spikes were collected at physiological maturity and weighed. For the dry weight of aerial parts, all parts above the soil line in each pot were collected, oven‐dried at 60°C for 48 h, and weighed. The relative water content (RWC), chlorophyll fluorescence, and chlorophyll content were measured at the time of sampling. The RWC was measured around midday of the sampling date following the method described by Turner (1981). For chlorophyll fluorescence, the flag leaf on the main stem was dark adapted for 0.5 h with leaf clips before using a pocket PEA chlorophyll fluorimeter (Hansatech Instruments Ltd, Norfolk, UK) for measuring chlorophyll fluorescence according to the ratio of variable fluorescence to maximum fluorescence (Fv/Fm). Chlorophyll content was measured by an SPAD‐502 Plus chlorophyll meter (Konica Minolta, Osaka, Japan) on the flag leaf of the main stem, and raw reads (SPAD values) were used as a comparison for chlorophyll content in tolerant and susceptible isolines. Comprehensive morphological and physiological information of NILs under WW and DS conditions can be found in our previous study (Nouraei et al., 2022).

### 2.3 Protein extraction

The flag leaf was finely ground with a mortar and a pestle under liquid nitrogen. Approximately 100 mg of finely ground sample was transferred to a 2 mL tube and thoroughly mixed with 300 μL of extraction buffer containing 125 mM Tris‐HCL pH 7.0, 7% (w/v) SDS, 5% (w/v) PVP‐40, 15 mM DTT, and Roche mini protease inhibitor cocktail (one tablet per 10 mL of extraction buffer). After centrifuging at 14,000 g for 300 s, 200 μL of supernatant was transferred to a new tube and precipitated by adding 800 μL methanol, 200 μL chloroform, and 600 μL distilled deionized water according to the chloroform/methanol method (Cao et al., 2021). After another centrifugation at 14,000 g at 4°C for 300 s, the upper aqueous phase was carefully removed, and the protein precipitated as a pellet by adding 800 μL methanol. Finally, the protein pellet was washed twice in 80% (v/v) acetone and stored at −80°C until further analysis.

### 2.4 Protein digestion and iTRAQ labeling

The protein pellets were resuspended in 0.5 M triethylammonium bicarbonate (pH 8.5) and 0.1% SDS and digested with trypsin at 37°C for 3 h. Samples were reduced with TCEP and alkylated with methyl methanethiosulfonate (MMTS) before a second digestion with trypsin at 37°C for 24 h (Islam et al., 2020). After centrifuging at 13,000 g for 600 s at room temperature, the peptide concentration was assayed by removing the supernatant, and 100 μg aliquot from each sample was removed for iTRAQ 8‐plex labeling according to the manufacturer's instructions (AB Sciex, Framingham, MA, USA). After labeling, samples from the same set were combined to make a pooled sample and desalted on Strata‐X 33 μm polymeric reversed phase columns (Phenomenex, Torrance, CA, USA).

### 2.5 Cation‐exchange chromatography (SCX) fractionation

Strong cation exchange chromatography (SCX) was performed for peptide separation with Agilent 1100 HPLC system equipped with a Zorbax C18 column (2.1 × 150 mm, 5 μm, 95 Å, Agilent Technologies, Palo Alto, CA, USA). The iTRAQ‐labeled peptide mixtures were eluted at a flow rate of 0.2 mL/min with a linear gradient of 20 mM ammonium formate, 2% ACN to 20 mM ammonium formate, and 90% ACN. Elution was monitored by measuring the absorbance at 280 nm, and fractions were collected every minute. A total of 95 × 1 min fractions were combined into 12 fractions, desalted on Strata‐X 33 μm columns, and vacuum dried prior to the liquid chromatography–mass spectrometry (LC–MS/MS) analysis.

### 2.6 LC–MS/MS analysis

Each fraction was resuspended in 0.1% formic acid and loaded on to an Acclaim PepMap 100 C18 LC Column, 2 μm particle size × 150 mm (Thermo Fisher Scientific, Waltham, MA, USA) running on a Thermo UltiMate 3000 nanoflow UHPLC system (Thermo Fisher Scientific). The mass spectrometry was analyzed by Q‐Exactive HF mass spectrometer (Thermo Fisher Scientific). MS1 spectra were collected with the following parameters: 120,000 resolution, scan range 300–1800 *m*/*z*, 3e6 automatic gain control (AGC), 30 ms maximum ion injection time (MIT), and data collected in profile. MS2 scans were conducted with the following parameters: 30,000 resolution, 150 ms MIT, 1e5 AGC, 1.6 *m*/*z* isolation window, loop count of 10, NCE of 28, 100.0 *m*/*z* fixed first mass, peptide match set to preferred, and data collected in centroid and at a dynamic exclusion of 45 s.

### 2.7 iTRAQ data analysis

MS/MS spectra were analyzed with ProteinPilot software version 5.0.2 (AB Sciex, Framingham, MA, USA) for protein identification and quantification using the Paragon algorithm (Shilov et al., 2007). The raw data were searched against the bread wheat cv. Chinese Spring (CS) curated reference protein sequence on the UniProt database (https://www.uniprot.org/proteomes/UP000019116) published by the International Wheat Genome Sequencing Consortium (IWGSC RefSeq v1.0). The database consisted of 130,673 predicted proteins and search parameters were: iTRAQ 8‐plex (peptide labeled) sample type, cysteine alkylation MMTS, trypsin digestion, *T. aestivum* FASTA database, and identification focus on biological modification, thorough the searching mode. Proteomics System Performance Evaluation Pipeline (PSPEP) Software, an add‐on function in ProteinPilot software, was used to estimate the global false discovery rate (FDR) on Paragon search results. Unique proteins with at least two unique peptides that were identified with >95% confidence (identification score >1.3) and an FDR of ≤1% were considered for further analysis. The mass spectrometry proteomics data have been deposited to the ProteomeXchange Consortium via the PRIDE (Perez‐Riverol et al., 2022) partner repository with the dataset identifier PXD036534.

### 2.8 Differential expression (DE) and bioinformatics analysis

The differential expression analysis was performed comparing the protein expression between TD_TC; SD_SC; TD_SD; and TC_SC through ProteinPilot software version 5.0.2 (https://sciex.com/products/software/proteinpilot‐software) (Shilov et al., 2007). Symbols are “C” for control (WW); “D” for DS; “T” for tolerant; and “S” for susceptible, respectively. Differentially expressed proteins (DEPs) were identified by using unpaired two‐tailed Student's *t*‐test with the fold change (FC) threshold of ≥1.3 for upregulation and ≤0.7 for downregulation; and a *p‐*value of < 0.05. The Venny v2.1 online Venn diagram generator (http://bioinfogp.cnb.csic.es/tools/venny/index.html) was used to create four‐way Venn diagrams (Oliveros, 2007–2015). The volcano plots were created in VolcaNoseR (https://huygens.science.uva.nl/VolcaNoseR/) using log2 of FCs and −log10 of *p*‐values (Goedhart & Luijsterburg, 2020). The ShinyGO 0.76 tool (http://bioinformatics.sdstate.edu/go/) was used for gene ontology (GO) enrichment analysis with an FDR cutoff of 0.05 and *T. aestivum* as the genetic background (Ge et al., 2020). The KEGG Orthology (KO) assignment and Kyoto Encyclopedia of Genes and Genomes (KEGG) mapping were conducted for identified DEPs in the KEGG database (https://www.kegg.jp/). For the KO assignment, the amino acid sequences of DEPs were BLAST in KOALA (KEGG Orthology and Links Annotation) tool (https://www.kegg.jp/blastkoala/) (Kanehisa et al., 2016). Then the KEGG Mapper tool (https://www.kegg.jp/kegg/mapper/) was used to construct KEGG pathways from a set of identified K numbers (Kanehisa & Sato, 2020). The PPI network was analyzed by using the combination of STRING database v11.5 (https://string‐db.org/) and the Cytoscape software v3.9.1 (https://cytoscape.org/) (Shannon et al., 2003; Szklarczyk et al., 2018). The CIRCOS software package v0.69‐9 (http://circos.ca/) was used for visualizing data in circos plots (Krzywinski et al., 2009). The MapChart v2.32 (https://www.wur.nl/en/show/mapchart.htm) mapped protein‐encoding genes on the 4BS (Voorrips, 2002).

### 2.9 In silico expression analysis of the identified genes

To provide more supportive information regarding the role of the identified genes in DS as well as other abiotic stresses, the expression of the candidate genes responsible for drought tolerance on 4BS was further investigated in the Wheat Expression Browser (expVIP, http://www.wheat‐expression.com) (Ramírez‐González et al., 2018) and the wheat multi‐omics database (WheatOmics, http://202.194.139.32/expression/wheat.html) (Ma et al., 2021).

### 2.10 Quantitative real‐time PCR (qRT‐PCR) assays

Total RNA was extracted from leaf samples using ISOLATE II RNA plan kit with on‐column DNase I treatment according to the manufacturer's instructions (Meridian Bioscience, Cincinnati, OH, USA). The PrimerQuest tool (https://sg.idtdna.com) was used for gene‐specific (exon–exon junction) primer design. The total RNA was reverse‐transcribed in a 20 μL reaction system by SensiFAST cDNA Synthesis Kit (Meridian Bioscience) and used for real‐time polymerase chain reaction (qRT‐PCR) on ABI 7500 Fast Real‐Time PCR (Applied Biosystems, Waltham, CA, USA) (Wang et al., 2021). The reactions were performed in 96‐well plates with a 25 μL reaction mixture (in each well) containing 10 μL SensiFAST SYBR Lo‐ROX (Meridian Bioscience). Actin was used as a reference gene for normalization between samples. Relative expression levels were calculated using the 2^−△△^ *^Ct^* method, and the average value of the three technical replications was considered for each of the three biological replications.

## 3 RESULTS

### 3.1 Morphological and physiological differences between near‐isogenic lines under drought stress

**FIGURE 1:**
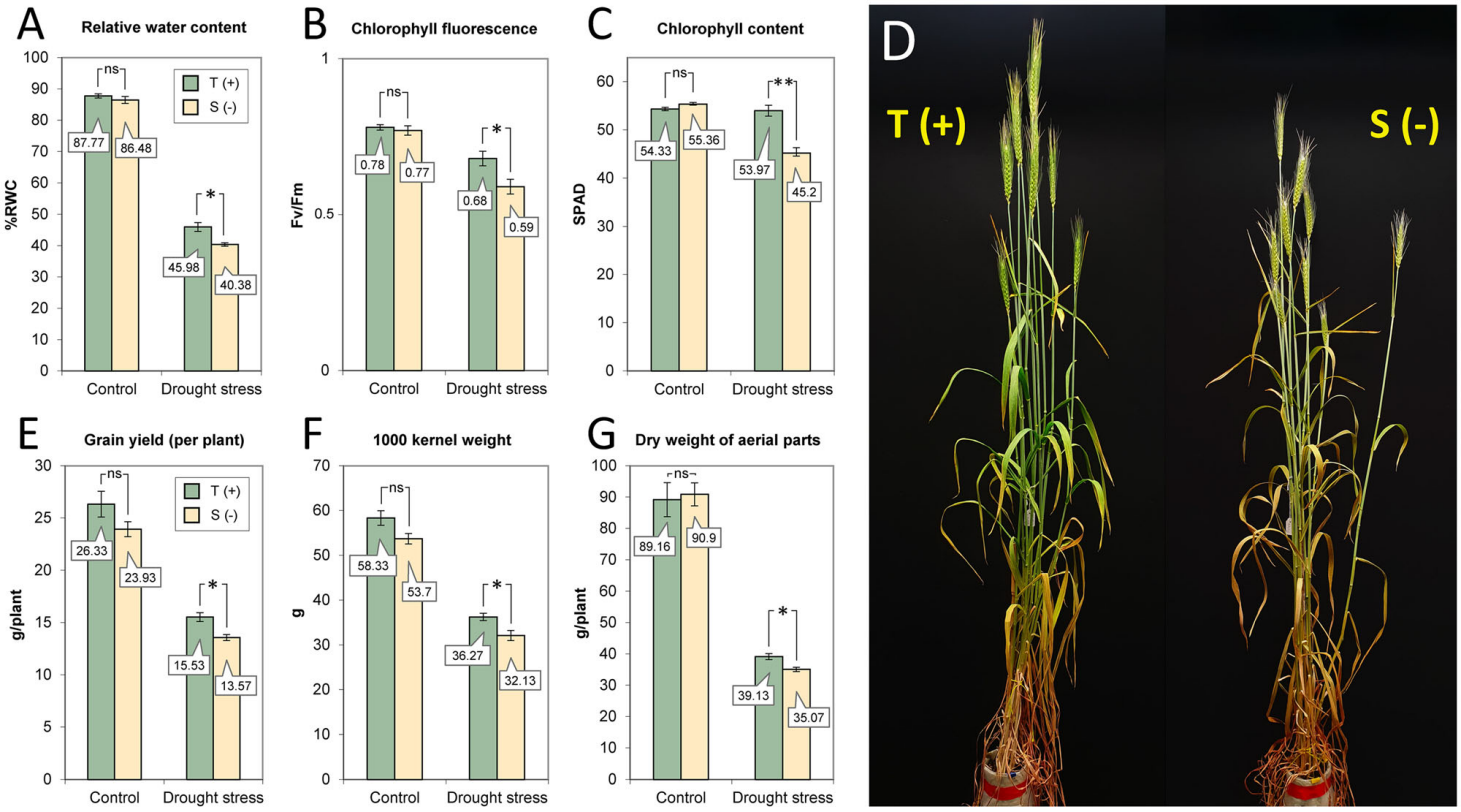
Morphological and physiological characteristics of drought tolerant and susceptible near‐isogenic lines (NILs) in wheat: (A) relative water content, (B) chlorophyll fluorescence, and (C) chlorophyll content of NILs in the well‐watered (Control) and after withholding water for 7 days (drought stress) at anthesis; (D) representative tolerant (T) and susceptible (S) plants 7 days after withholding water at anthesis; (E) grain yield, (F) 1000‐kernel weight, and (G) dry weight of aerial parts at maturity after withholding water at anthesis for 7 days. Data represents means ± one standard error (*n* = 3 biological replicates). ns: nonsignificant, *p < 0.05, and **p < 0.01 by Student's *t*‐tests.

No significant differences were observed between NILs for phenotypic and physiological measurements in the WW (control) treatment, as both lines maintained intact plant architecture. Seven days after anthesis, the leaf RWC around midday was 87.8% and 86.5% (Figure 1A), the chlorophyll fluorescence was 0.78 and 0.77 (Figure 1B), and the chlorophyll content (SPAD reading) was 54.3 and 55.4 (Figure 1C), all nonsignificantly different, in the drought tolerant and drought susceptible isolines, respectively. However, there were significant differences in the performance of the two isolines under post‐anthesis DS. Both isolines showed some common signs of stress, including wilting, yellowing, and drying leaves, which indicated that they were both subjected to moderately severe water shortage (Figure 1D). This was confirmed by the measured values of RWC and chlorophyll content and fluorescence: In the DS treatment, RWC fell to 46.0% and 40.4% (Figure 1A), the chlorophyll fluorescence values were 0.68 and 0.59 (Figure 1B), and the chlorophyll content values were 54.0 and 45.2 (Figure 1C), all significantly (*p* < 0.05) lower in the drought susceptible than drought tolerant isoline. Moreover, the leaves on the susceptible isoline were more yellow and were more shriveled, whereas the tolerant isoline exhibited less phenotypic changes by keeping expanded green leaves with slight yellowing at the leaf tips and intact plant architecture (Figure 1D).

The 7 days of increasing DS significantly (*p* < 0.05) decreased grain yield, 1000‐kernel weight, and dry weight of aerial parts at maturity. Grain yield was reduced from 25 g per plant in the control treatment (WW) to 15.5 and 13.6 g per plant in the DS treatment in T and S isolines, respectively (Figure 1E). The 1000‐kernel weight decreased from 56 g for T and S isolines in the WW treatment to 36.3 and 32.1 g in the DS treatment in T and S isolines, respectively (Figure 1F), whereas DS reduced the dry weight of aerial parts from 90 g per plant in the WW treatment to 39.1 and 35.1 g per plant in the T and S isolines, respectively (Figure 1G).

### 3.2 Primary data analysis and protein detection by iTRAQ in near‐isogenic lines

A total number of 77,078 spectra matched to known spectra, 27,997 distinct peptides, and 3189 proteins were identified using the ProteinPilot software v5.0.2. Among the 3,189 identified proteins, molecular weight of 49 (1.53%) was <10 kDa, 2639 (82.75%) were 10–70 kDa, 296 (9.29%) were 70–100 kDa, and 205 (6.42%) were >100 kDa (Figure S1A). Additionally, 589 (18.5%) of the proteins had only 1 identified unique peptide, and the remaining 2600 (81.53%) were detected with at least 2 unique peptides (Figure S1B). Peptide length distribution showed that 88.4% of the peptides had lengths between 5 and 20 amino acids (Figure S1C). Protein sequence coverage for 81.7% of the proteins was less than 40% (Figure S1D).

### 3.3 Differentially expressed proteins (DEPs) in different pairwise comparisons

**FIGURE 2:**
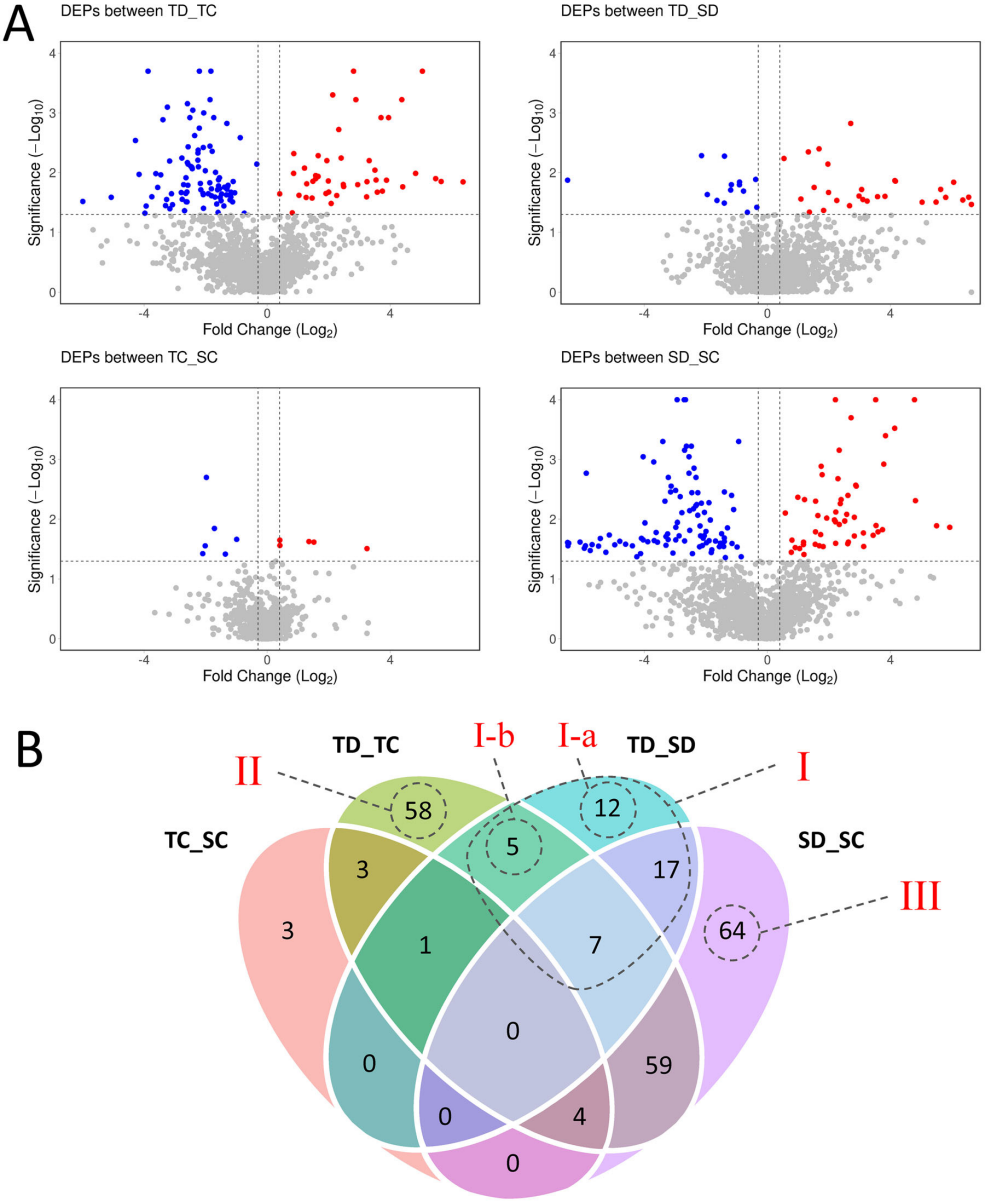
(A) Volcano plots and (B) Venn diagram for differentially expressed proteins (DEPs) identified in the four pairwise comparisons. In part (A), *X*‐axis indicates the log2 of fold change and *Y*‐axis represents −log10 of the *p*‐value. Upregulated proteins are red and downregulated proteins are blue; gray proteins were not significantly changed. In part (B), area I represents the DEPs identified in TD_SD excluding TC_SC comparison; area I‐a includes DEPs found specifically in TD_SD, and I‐b represents those shared between TD_SD and TD_TC; areas II and III include drought‐responsive proteins specific to the tolerant and susceptible isolines, respectively. Symbols are “T” for tolerant and “S” for susceptible isoline; “C” for control and “D” for drought stress treatment.

To investigate the protein change in the NILs in the DS treatment, comparative proteomic analyses were conducted. This included four pairwise comparisons between tolerant (T) and susceptible (S) isolines in the WW and DS treatments. The significant DEPs in the four comparisons and their regulation are displayed in volcano plots (Figure 2A). In the WW treatment, 11 DEPs were observed between the T and S isolines (TC_SC), including 5 upregulated and 6 downregulated proteins (Table 1). In the DS treatment, a total number of 42 DEPs were identified between the T and S isolines (TD_SD). Of these DEPs, 28 proteins had higher expression, and 14 had lower expression in the T compared to the S isoline. In the T isoline, 137 proteins showed differential expression in comparison after and before DS (TD_TC), 45 of these DEPs were upregulated, and 92 were downregulated in the DS treatment. In the S isoline, 151 DEPs were identified between stress and nonstress conditions (SD_SC). Of these DEPs, 54 proteins were upregulated, and 97 were downregulated in the DS treatment (Table 1).

**TABLE 1:** Number of differentially expressed proteins (DEPs) identified in each comparison group.

Comparisons^a^	Upregulated^b^	Downregulated^c^	Total^d^
TC_SC	5	6	11
TD_SD	28	14	42
TD_TC	45	92	137
SD_SC	54	97	151

^a^ Different comparison groups; TC, tolerant isoline in the well‐watered (control) treatment; TD, tolerant isoline in the drought stress treatment; SC, susceptible isoline in the control treatment; SD, susceptible isoline in the drought stress treatment; the underscore sign between two isolines indicates the comparison between the two treatments.

^b^ Upregulated: proteins with increased abundance in the former isoline in comparison to the latter isoline.

^c^ Downregulated: proteins with reduced abundance in the former isoline in comparison to the latter isoline.

^d^ Total: total number of differentially expressed proteins in each comparison group.

### 3.4 Combinations of comparisons in respect of drought tolerance

A Venn diagram illustrates the combinations of the four pairwise comparisons between T and S isolines in the WW and DS treatments, showing the impact of genotype or treatment in protein expression (Figure 2B). The most important comparison that represents proteins responsible for drought tolerance (Area I) is the comparison between T and S isolines in the DS treatment (TD_SD), excluding the DEPs between T and S isolines in the WW treatment (TC_SC). Of the 41 DEPs in this comparison (Table S1), 12 proteins that were found specifically in the TD_SD comparison (Area I‐a), and 5 proteins that were shared between TD_SD and TD_TC (Area I‐b) are considered the most important candidate proteins responsible for drought tolerance (Figure 2B). Area II represents the DEPs of TD_TC that were drought‐responsive proteins specific to the drought‐tolerant isoline. Area III represents the DEPs of SD_SC that were drought‐responsive proteins specific to the susceptible isoline (Figure 2B).

### 3.5 Expression patterns and locations of DEPs

**FIGURE 3:**
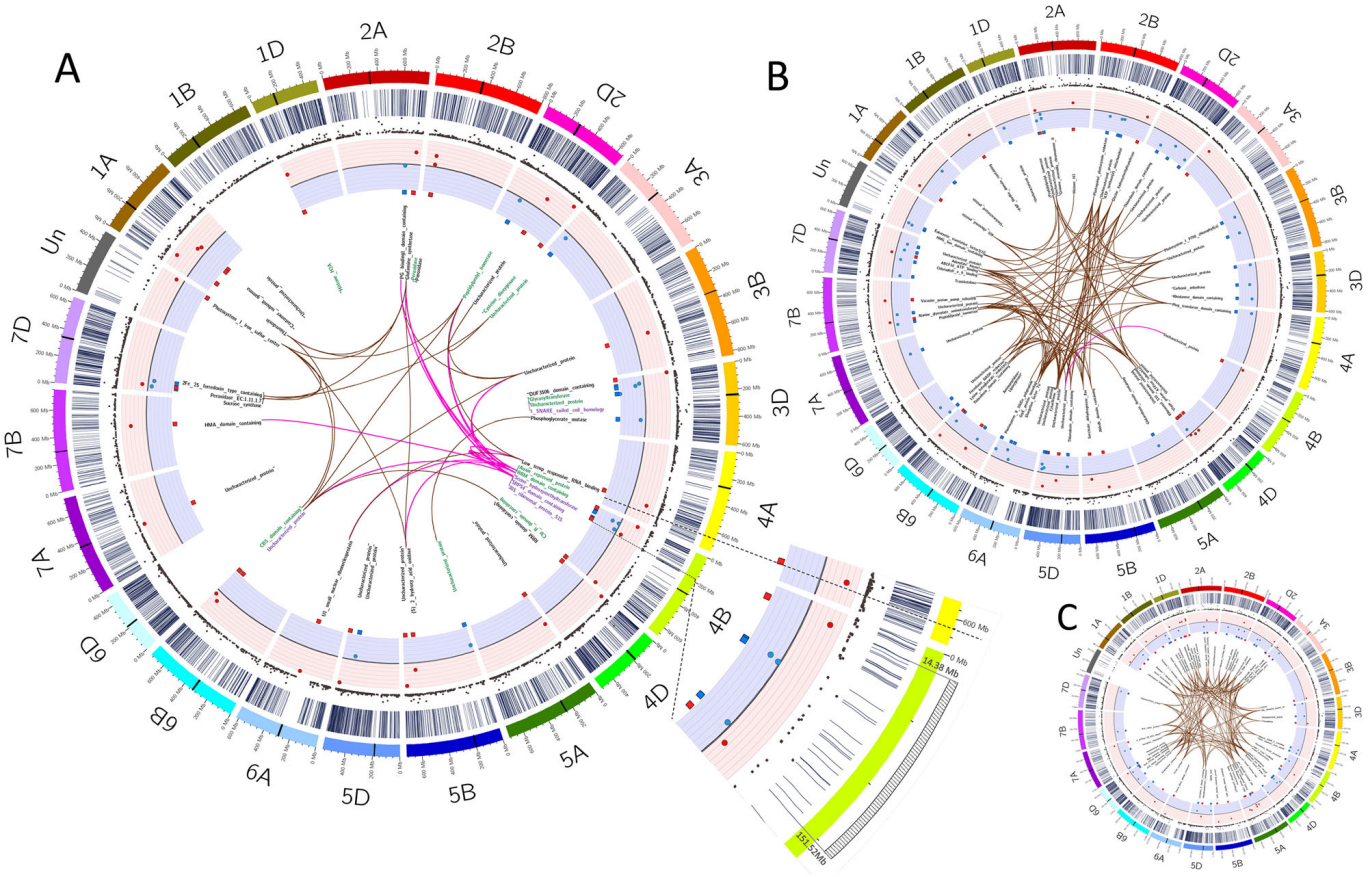
Circos plots showing the distribution of (A) 41 proteins responsible for drought tolerance (B) 58 drought‐responsive proteins specific to the drought‐tolerant isoline, and (C) 64 drought‐responsive proteins specific to the drought‐susceptible isoline mapped to different chromosomes of wheat. Circles: (1) chromosomes (thick lines are centromeres); (2) all identified proteins; (3) peptide count for all identified proteins; (4) scatter plot showing log2 of fold changes for differentially expressed proteins (DEPs) in colored circles, red color represents upregulation and blue represents downregulation; (5) red and blue squares representing regulation and location of DEPs; (6) text plot showing name of DEPs, in part (A), green text represents 12 proteins specific to TD_SD comparison (Area I‐a), and purple text represents 5 proteins shared between TD_SD and TD_TC (Area I‐b); (7) link tracks illustrating interaction between proteins according to protein–protein interaction analysis through STRING database (https://string‐db.org/), pink links representing interactions of proteins located on 4BS with other proteins. Un: unknown chromosome, including mitochondria and chloroplast proteins.

The location of encoding genes, expression pattern, name, and PPI of DEPs of areas I (including [I‐a] and [I‐b]), II, and III (Figure 2B) are illustrated as circos plots (Figure 3A–C and Figures S2–S4). Area I had 41 DEPs, 27 upregulated, and 14 downregulated (according to the TD_SD comparison) located on all chromosomes except 1B and 6D (Figure 3A; Figure S2 and Table S1). Of the 12 proteins in area I‐a, 6 proteins were upregulated, and 6 were downregulated in the TD_SD comparison. Two of these proteins were coded by genes on 4BS, one on 4BL, and the rest on 1D, 2B, 2D, 3A, 3D, 5B, and 6B (Figure 3A; Figure S2 and Table 2). Of the five proteins in area I‐b, two were upregulated and three were downregulated in the TD_SD comparison with three of them on 4BS, one on 3D, and one on 6B (Figure 3A; Figure S2 and Table 3). Of the 58 DEPs that were identified in area II, 19 proteins were upregulated, and 39 were downregulated in the TD_TC comparison. One of these proteins was located on 4BS, and the rest were spread on the other 20 wheat chromosomes (Figure 3B; Figure S3 and Table S2). Area III had 64 DEPs; a total of 30 upregulated and 34 downregulated that were located on all 21 chromosomes (Figure 3C; Figure S4 and Table S3).

**TABLE 2:** Proteins responsible for drought tolerance specifically found differentially expressed between the tolerant and sensitive isolines in the drought stress treatment (TD_SD).

N	Protein ID^a^	Gene ID^b^	Protein name^c^	Coverage (%)^d^	Peptides (95%)^e^	Fold change^f^	*p* value^g^	Pathways^h^
1	A0A3B6DQG3	*TraesCS2D02G541600*	Cysteine dioxygenase	12.2	2	49.5472	0.0191	–
2	A0A3B6IKM2	*TraesCS4B02G020300*	RRM domain‐containing protein	69.9	16	17.6411	0.0136	–
3	A0A3B5ZPX9	*TraesCS1D02G039900*	Histone H2A	53.9	18	8.5946	0.0282	–
4	A0A3B6IYI4	*TraesCS4B02G393100*	Clp R domain‐containing protein	24.5	12	6.5311	0.0015	–
5	Q43212	*TraesCS2B02G125200*	Peroxidase	50.6	21	6.3623	0.0359	Phenylpropanoid biosynthesis
6	A0A3B6PSD3	*TraesCS6B02G432300*	CBS domain‐containing protein	28	4	2.1281	0.0277	–
7	A0A0M3LQU0	*TraesCS4B02G070300*	Auxin‐repressed protein (ARP1)	42.6	2	0.7872	0.0381	–
8	A0A3B6DA29	*TraesCS2D02G208600*	Peptidylprolyl isomerase	46.9	29	0.5838	0.0204	–
9	A0A3B6GMS1	*TraesCS3D02G120200*	Glycosyltransferase	6.2	5	0.4372	0.0196	Biosynthesis of various plant secondary metabolites
10	A0A3B6EFE3	*TraesCS3A02G103900*	Uncharacterized protein	14.3	5	0.3787	0.0324	Starch and sucrose metabolism
11	A0A3B6LDJ7	*TraesCS5B02G016100*	Uncharacterized protein	68.1	24	0.3213	0.0293	–
12	A0A3B6GPR1	*TraesCS3D02G136800*	Uncharacterized protein	50.8	17	0.2268	0.0052	–

^a^ Protein ID: unique protein accession number in the UniProt database (https://www.uniprot.org).

^b^ Gene ID: unique identifying number of the gene corresponding to the protein according to the IWGSC RefSeq v1.0 wheat assembly (https://wheat‐urgi.versailles.inra.fr).

^c^ Protein name: recommended protein name according to the UniProt database.

^d^ Coverage (%): the percentage of matching amino acids from identified peptides divided by the total number of amino acids in the sequence.

^e^ Peptides (95%): number of peptides that have been identified associated with a protein with at least 95% confidence.

^f^ Fold change: the average ratio for the protein as the relative change between the two isolines; ratios above 1 mean proteins were upregulated and ratios below 1 mean proteins were downregulated.

^g^ *p*‐value: standard statistical metric to assess whether changes in protein expression are real or not; proteins with *p* < 0.05 were selected as significantly changed proteins.

^h^ Pathways: biological pathways in which the identified protein was found to be involved according to Kyoto Encyclopedia of Genes and Genomes (KEGG) database (https://www.kegg.jp/); TD, tolerant isoline in the drought stress treatment; SD, susceptible isoline in the drought stress treatment.

Abbreviation: RRM, RNA recognition motif.

**TABLE 3:** Proteins responsible for drought tolerance as differentially expressed proteins (DEPs) between the tolerant and sensitive isolines in the drought stress treatment (TD_SD) that were also differentially expressed in the tolerant isoline in the drought stress and control (well‐watered) treatments (TD_TC).

						TD_SD		TD_TC		
N	Protein ID^a^	Gene ID^b^	Protein name^c^	Coverage (%)^d^	Peptides (95%)^e^	Fold change^f^	*p* value^g^	Fold change	*p* value	Pathways^h^
1	A0A3B6PQC1	*TraesCS6B02G371500*	Uncharacterized protein	39.1	4	4.7724	0.0293	0.0636	0.0478	–
2	A0A341RD03	*TraesCS4B02G124900*	30S ribosomal protein S15	47.1	3	2.5155	0.0045	0.248	0.0093	Ribosome
3	A0A3B6IRC2	*TraesCS4B02G117900*	SRP54 domain‐containing protein	31.4	17	0.6368	0.0463	0.3086	0.0257	Protein export
4	A0A0C4BJE5	*TraesCS4B02G069300*	Serine hydroxymethyltransferase	66.9	123	0.2583	0.0233	0.2394	0.001	Glyoxylate and dicarboxylate metabolism
5	A0A3B6GUC6	*TraesCS3D02G180500*	t‐SNARE coiled‐coil homology domain‐containing protein	21.7	2	0.0111	0.0134	82.414	0.0143	SNARE interactions in vesicular transport

^a^ Protein ID: unique protein accession number in the UniProt database (https://www.uniprot.org).

^b^ Gene ID: unique identifying number of the gene corresponding to the protein according to the IWGSC RefSeq v1.0 wheat assembly (https://wheat‐urgi.versailles.inra.fr).

^c^ Protein name: recommended protein name according to the UniProt database.

^d^ Coverage (%): the percentage of matching amino acids from identified peptides divided by the total number of amino acids in the sequence.

^e^ Peptides (95%): number of peptides that have been identified to be associated with a protein with at least 95% confidence.

^f^ Fold change: the average ratio for the protein as the relative change between the two isolines; ratios above 1 mean proteins were up‐regulated and ratios below 1 mean proteins were down‐regulated.

^g^ *p*‐value: standard statistical metric to assess whether changes in protein expression were real or not; proteins with *p* < 0.05 were selected as significantly changed proteins.

^h^ Pathways: biological pathways in which the identified protein was found to be involved according to the Kyoto Encyclopedia of Genes and Genomes (KEGG) database (https://www.kegg.jp/); TC, tolerant isoline in the well‐watered (control) treatment; TD, tolerant isoline in the drought stress treatment; SC, susceptible isoline in the control treatment; SD, susceptible isoline in the drought stress treatment.

### 3.6 Gene ontology (GO) enrichment analysis of the important DEPs for drought tolerance

**FIGURE 4:**
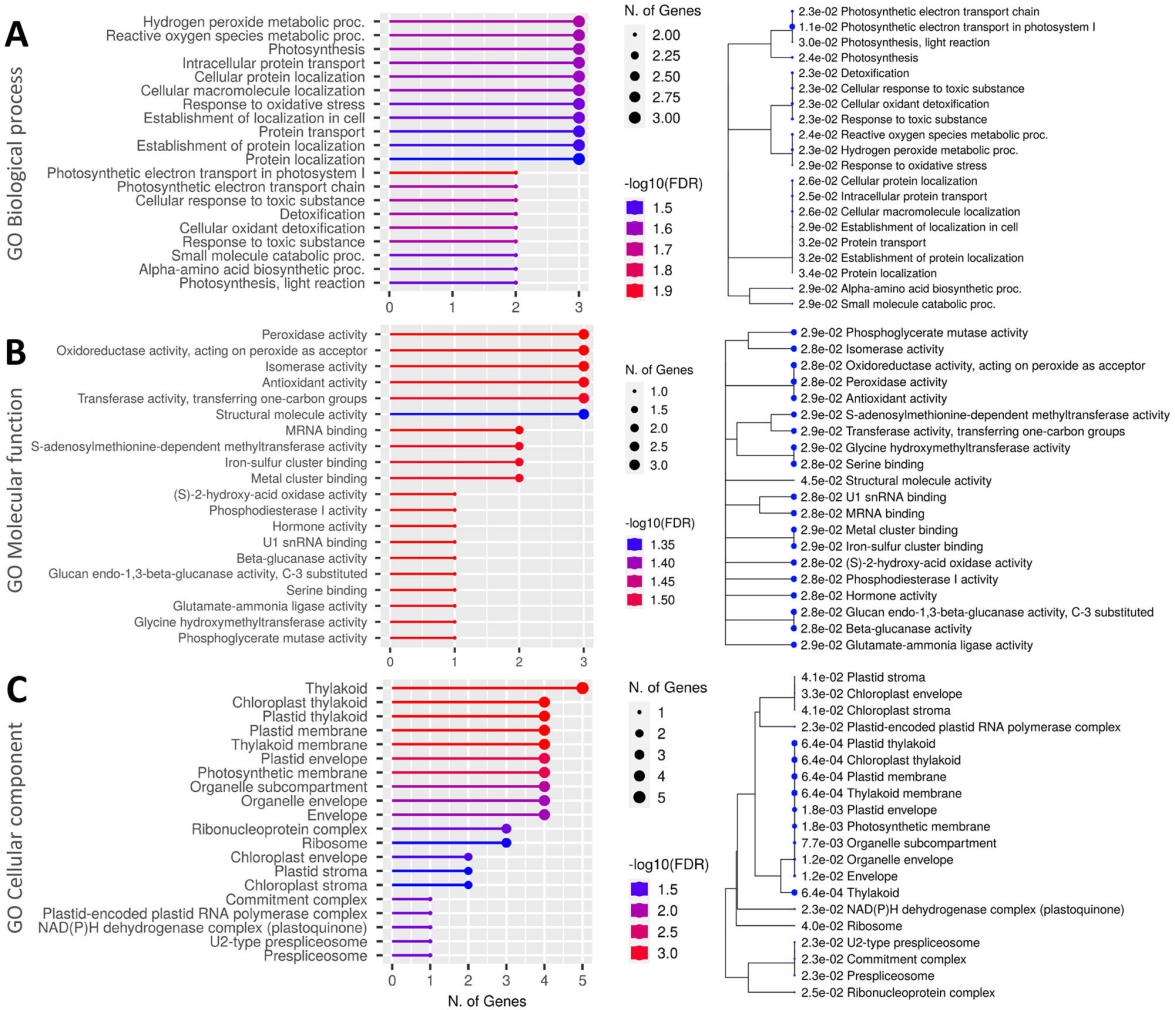
Gene ontology (GO) enrichment analysis of proteins responsible for drought tolerance. Lollipop charts on left showing top enriched pathways in (A) biological processes, (B) molecular functions, and (C) cellular components with the false discovery rate (FDR) < 0.05. *X*‐axis represents the number of genes (proteins); *Y*‐axis displays the functional categories. Hierarchical clustering trees on right summarize the correlations among significant enriched pathways listed on the left. Pathways with many shared genes are clustered together, and bigger dots indicate more significant *p*‐values.

GO enrichment analysis was conducted to better understand the different functions and processes in which the identified proteins are involved. The biological processes (Figure 4A), molecular functions (Figure 4B), and cellular components (Figure 4C) of the proteins responsible for drought tolerance (Area I of Figure 2B) are presented. According to the biological process analysis, most of the proteins were enriched in hydrogen peroxide metabolic processes, ROS metabolic processes, photosynthesis, intracellular protein transport, cellular protein localization, cellular macromolecule localization, and responses to oxidative stress (Figure 4A). Regarding their molecular functions, the identified proteins were mostly enriched in peroxidase, oxidoreductase, isomerase, antioxidant activity, transferase, and structural molecular activities (Figure 4B). Most of the proteins were enriched in the thylakoid, thylakoid membrane, plastid membrane, plastid envelope, chloroplast stroma, ribosome, and pre‐spliceosome cellular components (Figure 4C).

Additionally, GO enrichment analysis was performed for the drought‐responsive proteins specific to the drought‐tolerant isoline (Area II in Figure 2B). Photosynthetic processes, peptide biosynthetic processes, translation, homeostatic processes, and ion transmembrane transport were the major enriched biological processes (Figure S5A). The major enriched molecular functions were the structural constituents of the ribosome, structural molecule, pyrophosphate, hydrolase, nucleoside triphosphatase, adenosine triphosphate (ATP)‐dependent, and oxidoreductase activities (Figure S5B). The major enriched cellular components were the thylakoids, the photosynthetic membranes, the organelle envelope, membrane protein complex, chloroplast stroma, ribosome, and the mitochondrion (Figure S5C).

### 3.7 Protein–protein interaction (PPI) and Kyoto Encyclopedia of Genes and Genomes (KEGG) pathway analysis

**FIGURE 5:**
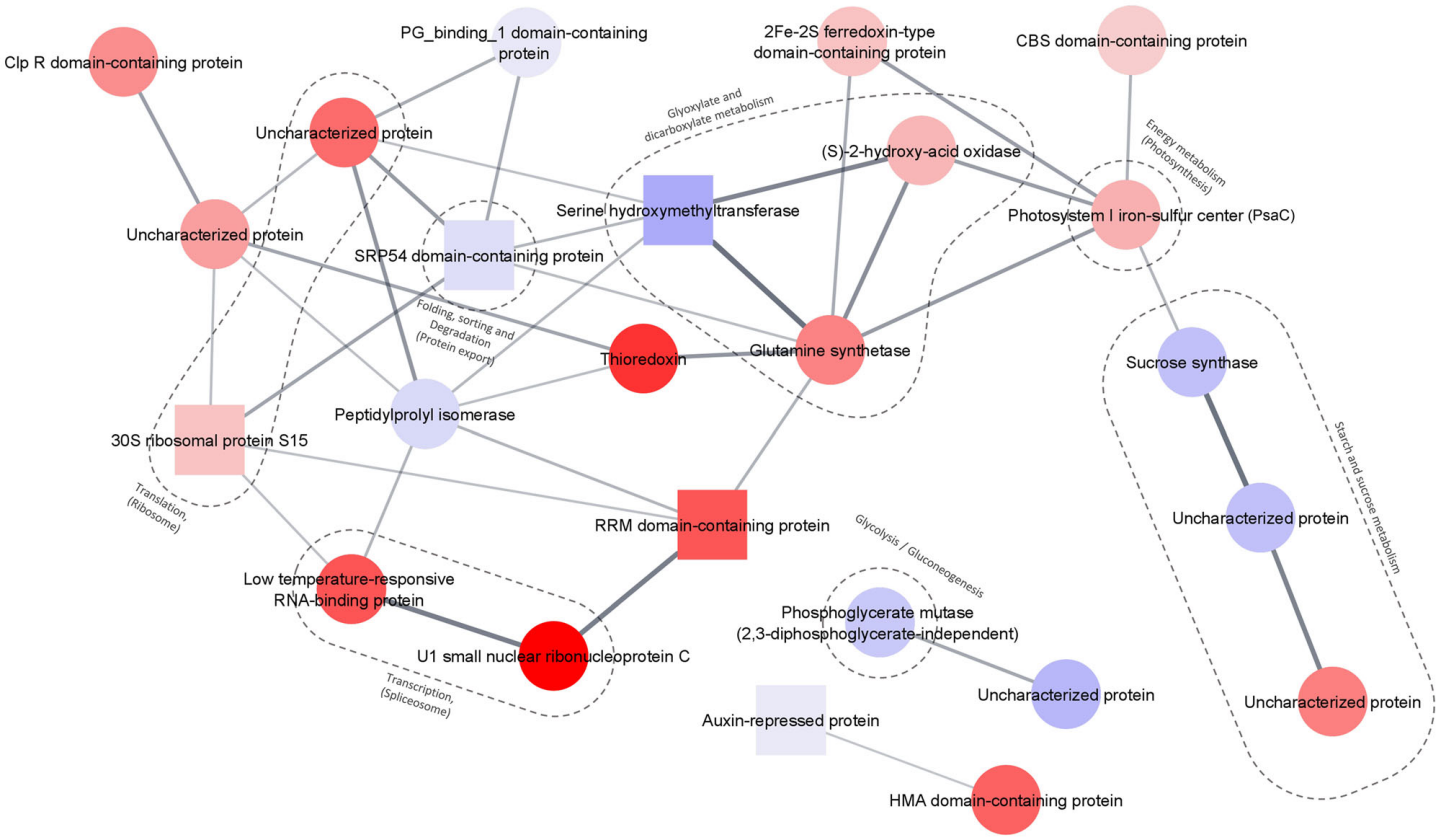
Protein interaction network of proteins responsible for drought tolerance. The network is predicted using the STRING database (https://string‐db.org/) and created in Cytoscape v3.9.1 (https://cytoscape.org/) with a confidence score higher than 0.5. Nodes representing proteins; color intensity corresponding to the fold change values; red proteins had higher, and blue proteins had lower abundance in tolerant than susceptible isolines after stress; line thickness represents the strength of the supporting data. Dotted lines showing pathway groups based on the Kyoto Encyclopedia of Genes and Genomes (KEGG) pathway analysis (https://www.kegg.jp/). Proteins with encoding genes located on the short arm of chromosome 4B (4BS) are shown as squares.

The majority of genes/proteins function in association and/or in coordination with a network of molecules rather than individual molecules. To provide a better understanding of the molecular mechanisms underlying drought tolerance, the PPI analysis was conducted through the STRING database. Additionally, to help better decipher the network, the important KEGG pathway groups were identified and highlighted on the PPI network. For the proteins responsible for drought tolerance (Area I of Figure 2B), connection between 24 (out of 41) proteins, including all 5 proteins located on 4BS, was identified (Figure 5). According to the KEGG pathway analysis, sucrose synthase (SuSy) in company with two uncharacterized proteins that were annotated as an ectonucleotide pyrophosphatase/phosphodiesterase family member 1/3 (ENPP) and 1,3‐beta‐glucan synthase by the KEGG database, play roles in starch and sucrose metabolism (Figure S6A); phosphoglycerate mutase (PGMi) participates in glycolysis/gluconeogenesis processes (Figure S6B); serine hydroxymethyltransferase (SHMT) (located on 4BS) along with (S)‐2‐hydroxy‐acid oxidase (HAO) and glutamine synthetase (GS) work in glyoxylate and dicarboxylate metabolism (Figure S7); the photosystem I (PSI) iron–sulfur center engages in energy metabolism through photosynthesis (Figure S8A); 54‐kDa protein subunit of the signal recognition particle (SRP54) domain containing protein is involved in protein export (Figure S8B); U1 small nuclear ribonucleoprotein (RNP) C and low temperature responsive RNA binding protein that has a role in RNA transcription during stress, functioning as spliceosome components in the transcription process (Figure S9A); and 30S ribosomal protein S15 (located on 4BS) and an uncharacterized protein (KEGG annotated as small subunit ribosomal protein S20) play roles in translation (Figure S9B). According to the PPI network, transcription and translation pathways show connection to each other; transcription and translation pathways are connected to glyoxylate and dicarboxylate metabolism through the SRP54 domain‐containing protein and RNA recognition motif (RRM) domain‐containing protein, respectively (both located on 4BS); the photosynthetic pathway (PSI iron–sulfur center) has interaction with the starch and sucrose pathway (SuSy) and glyoxylate and dicarboxylate metabolism pathways (GS and (S)‐2‐hydroxy‐acid oxidase). Auxin‐repressed protein (ARP) (located on 4BS) and heavy metal–associated domain‐containing protein are also associated with each other (Figure 5).

PPI analysis of the specific DEPs to the drought‐tolerant isoline (Area II in Figure 2B) linked 46 (out of 58) proteins, including 1 4BS protein, to each other (Figure S10). According to the KEGG pathway analysis, glycine, serine, and threonine metabolism with alanine–glyoxylate aminotransferase, aldehyde domain‐containing protein, and glycine hydroxymethyltransferase (Figure S11); translation with 40S ribosomal protein, ribosomal S10 domain‐containing protein, and two uncharacterized proteins (Figure S12A); oxidative phosphorylation with a V‐type proton ATPase subunit, ATP synthase subunit d, vacuolar proton pump subunit B, and succinate dehydrogenase (Figure S12B); photosynthesis with Photosystem I P700 chlorophyll *a* apoprotein A1, photosystem II 10 kDa polypeptide, cytochrome f, plastoquinol–plastocyanin reductase, chlorophyll *a*–*b* binding protein, ferredoxin‐NADP reductase, and an uncharacterized protein (Figure S13A,B) were identified pathways with the highest number of proteins. All proteins in the photosynthesis pathway were downregulated, and all proteins in glycine, serine, and threonine metabolism were upregulated. The comprehensive connection between these pathways and the rest of the proteins has been observed in the PPI network (Figure S10).

### 3.8 Quantitative real‐time RT‐PCR (qRT‐PCR) analysis

**FIGURE 6:**
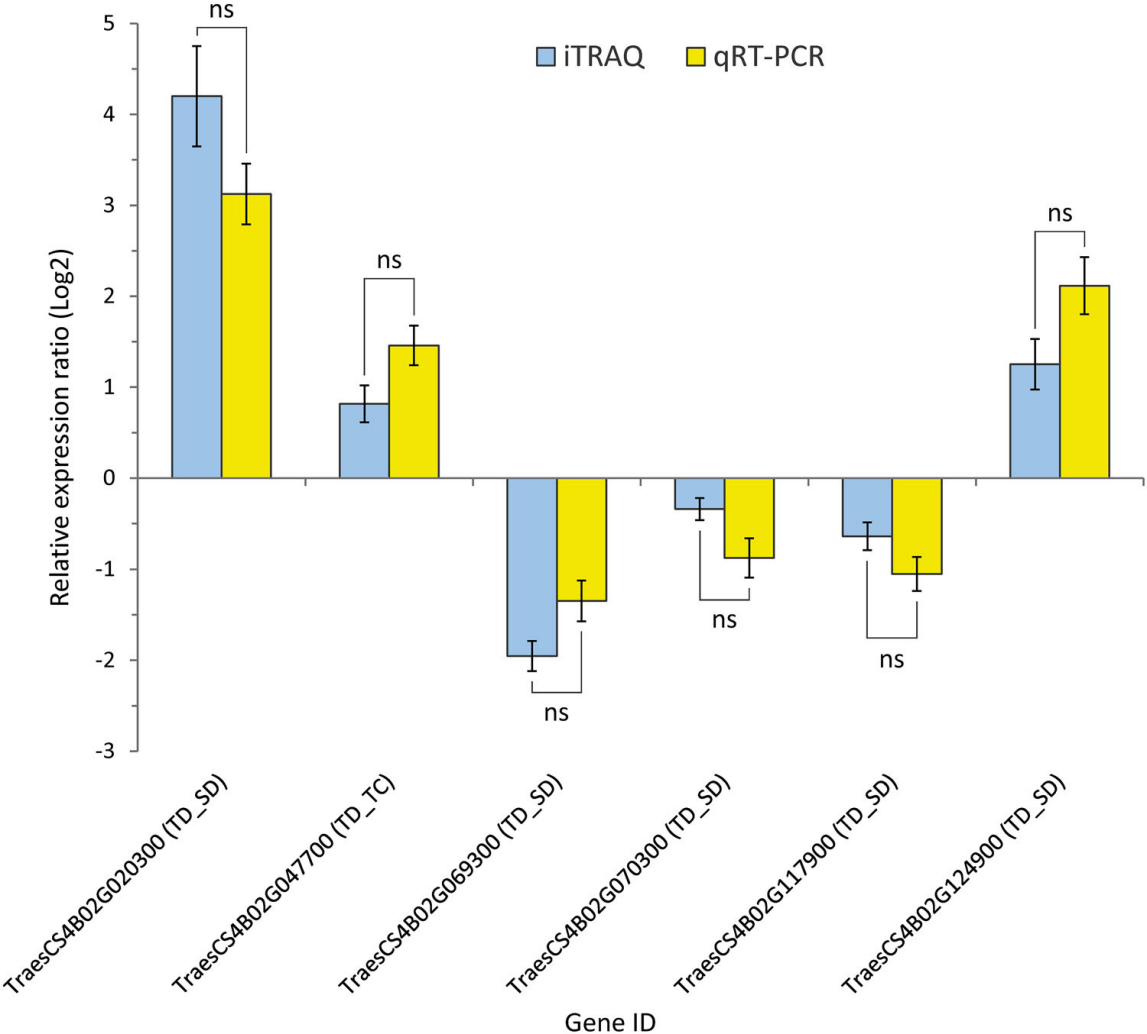
Confirmation of isobaric tags for relative and absolute quantitation (iTRAQ) results by quantitative real‐time polymerase chain reaction (qRT‐PCR). The *X*‐axis shows six candidate genes responsible for drought tolerance on the short arm of chromosome 4B. The *Y*‐axis represents log2 of the relative expression ratio (log2 of fold change) for iTRAQ and qRT‐PCR analyses. Gene IDs were adapted from the IWGSC RefSeq v1.0 wheat assembly (https://wheat‐urgi.versailles.inra.fr). Bars represent means ± standard error (*n* = 3 biological replicates). ns: nonsignificant by Student's *t*‐tests. Symbols are “T” for tolerant and “S” for susceptible isoline; “C” for control and “D” for drought stress treatment.

To confirm the results of iTRAQ analysis, a supporting experiment was conducted with qRT‐PCR for the six candidate genes responsible for drought tolerance on 4BS (Table S4). According to the qRT‐PCR results, the gene expressions for all six genes were in agreement with the iTRAQ protein expression (Figure 6). A correlation coefficient (*r*) of 0.92 was obtained between qRT‐PCR and iTRAQ FCs. These results showed high consistency between gene expression and protein abundance for the identified candidate genes and confirmed the reliability of the iTRAQ findings (Figure S14).

## 4 DISCUSSION

The imposition of DS by withholding water for 7 days from anthesis significantly reduced RWC by ∼50% and resulted in a reduction of ∼18% in chlorophyll fluorescence and ∼10% in the chlorophyll content of the flag leaf. It also resulted in a decrease in the dry weight of aerial parts (grain plus straw) by 56% and 61% and a reduction in grain yield by 38% and 46% in the T and S isolines, respectively. Grain size (1000‐kernel weight) was reduced by 35% in the T isoline and 43% in the S isoline suggesting that the main effect of the drought treatment was on grain size rather than the number of grains. Thus, the tolerant isoline that carried the positive allele for *qDSI.4B.1* QTL from the wheat cultivar C306 was less negatively affected than its susceptible counterpart by maintaining higher grain yield, 1000‐kernel weight, and dry weight of areal parts under stress condition. This confirmed stress imposition was effective, and the contrasting gene alleles in the isogenic lines were functioning.

To better understand the mechanism of drought tolerance in wheat at anthesis, the comparison between isolines under both control and stress conditions was conducted at the same developmental stage of 7‐day post‐anthesis to make sure the identified proteins and pathways are drought‐related rather than developmental stage–related. The iTRAQ proteomics analysis and following classification of identified DEPs assigned proteins to three important groups I, II, and III (Figure 2B). Due to higher importance, we mainly focused on the 41 proteins responsible for drought tolerance (Area I) and the only protein among the 58 drought‐responsive proteins specific to the drought‐tolerant isoline that had an encoding gene located on the 4BS chromosome arm (Area II).

### 4.1 Proteins responsible for drought tolerance

Five of the 41 proteins responsible for drought tolerance (Area I) had encoding genes located on the 4BS chromosome arm where the *qDSI.4B.1* QTL is located. Of these five, two proteins of RRM domain‐containing protein and ARP1 were among the DEPs specifically found between the tolerant and sensitive isolines in the DS treatment (Area I‐a) (Figure 2B and Table 2), and three proteins of 30S ribosomal protein S15, SRP54 domain‐containing protein, and SHMT were commonly shared DEPs between the TD_SD and TD_TC comparisons (Area I‐b) (Figure 2B and Table 3).

SuSy is a key enzyme in sucrose metabolism that catalyzes the reversible conversion of sucrose to uridine diphosphate glucose and fructose (Baud et al., 2004). Cofactor‐independent PGMi is an enzyme that catalyzes the reversible conversion of 3‐phosphoglycerate (3PG) to 2‐phosphoglycerate through the process of glycolysis (Zhao & Assmann, 2011). Sucrose and glycolysis metabolism (Figure S6A,B) are two important metabolic pathways in carbohydrate metabolism that contributes to the production of energy and primary and secondary metabolites (Cramer et al., 2013). Photosynthesis and carbohydrate metabolism are two of the most likely processes affected by DS in plants (Xue et al., 2008). However, changes in proteins and enzymes of carbohydrate metabolism were not always uniform. In this study, under stress condition, SuSy and PGMi were both downregulated in the tolerant isoline. In the proteomic analysis of *Brachypodium distachyon* roots and leaves, many DEPs related to energy metabolism were downregulated in leaves but upregulated in roots in response to DS (Bian et al., 2017). In rice, DS significantly reduced the activity of SuSy in leaves (Wang et al., 2022). In contrast, Du et al. (2020) reported enhanced SuSy enzyme activity in soybean leaves and roots under DS conditions, and Pan et al. (2016) reported the upregulation of PGM in drought‐tolerant lines of *Lolium multiflorum*. The reduction in the expression of enzymes involved in the glycolysis and sucrose pathways might be a mechanism to accumulate sugar as an osmolyte for drought adaptation by osmotic adjustment (Morgan, 1980; Turner, 2018) or source of energy for recovery from stress, whereas an increase in these enzymes could provide more energy for stress defense when photosynthesis is inhibited (Echevarría‐Zomeño et al., 2009).

Glyoxylate and dicarboxylate metabolism, including SHMT, HAO, and GS, is one of the main pathways responsible for drought tolerance (Figure S7). Glyoxylate and dicarboxylate metabolism has an important role in improving stress tolerance in plants by balancing metabolic disorders and transporting energy (Xu et al., 2018). In stress condition, HAO and GS were upregulated, and SHMT was downregulated in the tolerant isoline. GS is the ATP‐dependent enzyme that catalyzes the synthesis of glutamine from glutamate and plays a key role in the nitrogen metabolic pathway (Yin et al., 2022). Drought stress, by enhancing glutamine as a proline precursor in the tolerant isoline, can lead to the accumulation of proline and enhance the cellular osmoregulatory capacity (Díaz et al., 2010). The overexpression of GS in wheat and tobacco has been reported to improve drought tolerance by enhancing proline, chlorophyll, sucrose, and scavenging of ROS (Yu et al., 2020). Additionally, GS, HAO, and SHMT are involved in the photorespiratory cycle that can reduce oxidative stress by removing toxic metabolites (Figure S7). Photorespiration is a process in plant metabolism that converts 2‐phosphoglycolate made by the oxygenase activity of ribulose‐1,5‐bisphosphate carboxylase/oxygenase (RUBISCO) to 3PG (Noctor et al., 2012). SHMT is a key photorespiratory enzyme that converts glycine to serine and is involved in the control of cell damage caused by abiotic stresses, such as drought, salt, high light, and in the hypersensitive defense response of plants (Liu et al., 2019). The reduction in SHMT may result in the accumulation of glycine, which in turn could be mobilized for other processes such as the biosynthesis of glutathione, an important stress‐induced molecule involved with the detoxification of ROS (Noctor et al., 2012). Overaccumulation of polyamines in *shm1‐1* mutants in *Arabidopsis* under abiotic stress has been reported, but the mechanism behind it is not clear (Liu et al., 2019). Polyamines effectively regulate oxidative stress by increasing the activity of various antioxidant enzymes (D. Chen et al., 2018).

The PSI iron–sulfur subunit PsaC is 1 of 13 subunits found in the PSI complex of most plants and algae (Figure S8A). PSI mediates the light‐driven electron transfer across the thylakoid membrane from plastocyanin in the thylakoid lumen to ferredoxin in the chloroplast stroma (Gao et al., 2018; Ozakca, 2013). It consists five subunits of PsaA, PsaB, PsaC, PsaI, and PsaJ that are encoded in the chloroplast and eight subunits of PsaD, PsaE, PsaF, PsaG, PsaH, PsaK, PsaL, and PsaN that are encoded in the nucleus (Ozakca, 2013). Subunit PsaC has two terminal FeS clusters, FA and FB, which along with PsaD and PsaE subunits form the stromal hump on the stromal side of the complex (Scheller et al., 2001). Several studies have shown that the activity of PSI is prohibited by abiotic stresses such as DS (Liu et al., 2018; Ozakca, 2013; Suzuki et al., 2021). However, in our study, the higher abundance of PsaC in the tolerant compared to the susceptible isoline could be associated with a better photoprotection mechanism and in turn be responsible for the higher performance of the tolerant isoline under DS (Zivcak et al., 2014).

The SRP54 was found to be downregulated in the tolerant isoline in the DS treatment (Figure S8B). The SRTP54 is a protein—RNA complex that in eukaryotic organisms consists of a 7S RNA and six proteins named according to their molecular weights (SRP9, 14, 19, 54, 68, and 72) (Luirink & Sinning, 2004). The SRP54 is involved in the protein export pathway by transporting newly synthesized proteins into or across the cell membrane (Zopf et al., 1990). The *chaos* mutant in *Arabidopsis* with an impaired chloroplast‐recognition particle (cpSRP43) coding gene, showed lower ascorbate levels and H_2_O_2_ production, and better photosynthetic performance, which, in turn, resulted in higher tolerance to photooxidative stress (Klenell et al., 2005). However, the information about the exact role of the SRP54 protein in stress tolerance is limited. The ARP was downregulated in the tolerant isoline compared to the susceptible isoline in the DS treatment. Auxin, as a growth‐stimulating phytohormone, controls plant growth and development as well as plant responses to various biotic and abiotic stresses (Emenecker & Strader, 2020). The increase in the abundance of APRs under DS has been previously reported in peanuts (Govind et al., 2009). In tobacco, the *geri1* mutant with a mutation in the encoding gene of ARP1, showed growth enhancement and impaired pathogen resistance associated with the promotion of the expression of growth‐promoting genes and repression of defense‐responsive genes (Zhao et al., 2014). Drought stress has been shown to increase the susceptibility to pathogens in chickpea (Sinha et al., 2019). Likewise in our previous transcriptomics study with the same NILs, we observed the downregulation of all the disease‐resistance genes in the tolerant isolines when subjected to DS (Nouraei et al., 2022). Although several ARPs responsive to various abiotic stresses have been reported, their function in stress adaptation is less well known (L. Wu et al., 2017).

Spliceosome is a large RNP complex that removes introns from a transcribed pre‐mRNA through a process called splicing (Figure S9A) (Will & Lührmann, 2011). Among splicing components, U1 small nuclear RNP (U1 snRNP) is the smallest subcomplex and plays an important role in the recognition of the 5′ splice site in the early stages of splicing (Chen et al., 2020). In *Arabidopsis*, U1 snRNP was shown to be essential for stress resistance by regulating the alternative splicing of pre‐mRNAs (de Francisco Amorim et al., 2018). Low temperature–responsive RNA binding protein was another upregulated protein, which has been reported to be involved in RNA transcription and modification during low‐temperature stress and abscisic acid (ABA) application (Dunn et al., 1996). PPI analysis showed the RRM‐containing protein with higher abundance in the tolerant isoline than sensitive isoline had interaction with transcription, translation, and glyoxylate and dicarboxylate metabolism pathways. RRMs are found in a variety of RNA‐binding proteins that are involved in many post‐transcriptional processes, such as the splicing, editing, export, degradation, and regulation of translation (Nowacka et al., 2019). The overexpression of the RRM‐containing protein AlSRG1 in transgenic tobacco plants increased the transcript levels of ROS‐scavenging genes and some stress‐related transcription factors, which resulted in higher salt and osmotic tolerance in transgenic compared to non‐transgenic plants (Ben Saad et al., 2018). In *Arabidopsis*, RRM motif–containing protein (RBM25) was shown to play a critical role in drought tolerance by engaging in pre‐mRNA splicing and ABA responses (Zhan et al., 2015). The 30S ribosomal protein S15 (RPS15) is a member of ribosomal proteins (RP) that were upregulated in the tolerant isoline under drought stress (Figure S9B). Abiotic stress in plants causes the accumulation of ROS, which necessitates the synthesis of new proteins to defend against ROS and replace damaged proteins (Salih et al., 2020). The RP proteins play integral roles in forming and stabilizing the ribosomal complex and regulating gene expression at the transcription and translation levels (Shiraku et al., 2021). Studies on rice (Moin et al., 2017), *Arabidopsis* (Ramos et al., 2020), and tobacco (Liu et al., 2014) demonstrated that RPs were involved in abiotic stress tolerance by maintaining protein biosynthesis.

### 4.2 Drought‐responsive protein specific to the drought‐tolerant isoline with encoding gene located on chromosome 4BS

Of the 58 drought‐responsive proteins specific to the drought‐tolerant isoline (Area II), one protein with an uncharacterized name had an encoding gene located on 4BS and was upregulated under stress in the tolerant isoline. According to the PPI analysis, this protein interacted with another uncharacterized protein on 5D, which in turn had interactions with other proteins involved in photosynthesis, oxidative phosphorylation, and glycine, serine, and threonine metabolism. The BLAST of this uncharacterized protein sequence (the one located on 4BS) against UniProtKB and Swiss‐Prot databases, found the dynamin‐related protein 1C (DRP1C) with 95.6% amino acid sequence similarity was the closest annotated protein to our target protein. The DRP1 protein subfamily consists of five members from A through E that are involved in plant cell expansion and cytokinesis (Kang et al., 2001). Konopka et al. (2008) reported the localization of DRP1C at the division plane in dividing cells of *Arabidopsis* root hairs. Additionally, they witnessed DRP1C recruitment in plasma membranes of expanding interphase cells, suggesting the possible role of DRP1C in the clathrin‐mediated endocytic traffic.

### 4.3 The *qDSI.4B.1* QTL interval on 4BS

**FIGURE 7:**
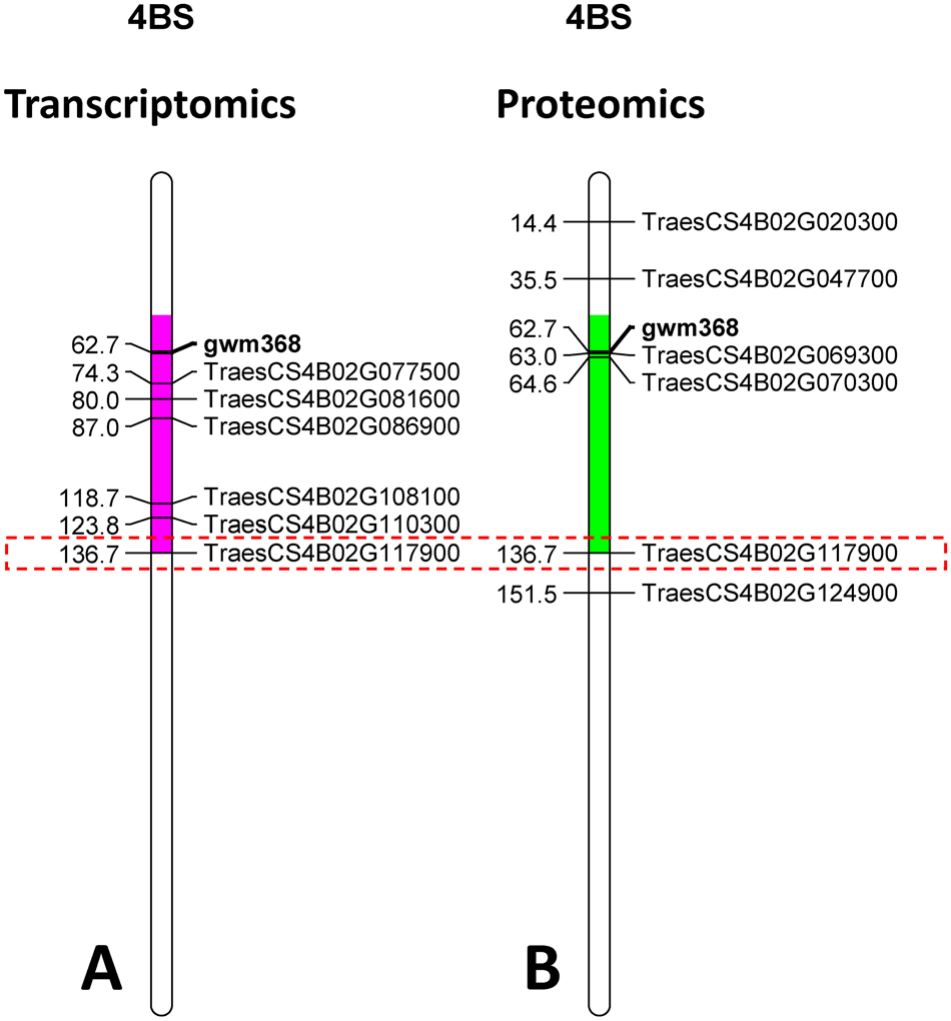
The distribution of responsible genes for drought tolerance on the short arm of chromosome 4B (4BS) according to (A) transcriptomic and (B) proteomic studies. The purple and green colors highlight the suggested location for *qDSI.4B.1* quantitative trait loci (QTL) based on the transcriptomics study. The gene of *TraesCS4B02G117900* was found in both studies, and *gwm368* (in **bold**) is the SSR marker used for developing the NILs. *Source*: The transcriptomic information was adapted from our previous study (Nouraei et al., 2022).

In the previous study, transcriptomic analysis was conducted on grain tissue of two pairs of NILs for the *qDSI.4B.1* QTL, and six candidate genes were found to be responsible for drought tolerance (Nouraei et al., 2022). These six genes included *TraesCS4B02G077500*, *TraesCS4B02G081600*, *TraesCS4B02G086900*, *TraesCS4B02G108100*, *TraesCS4B02G110300*, and *TraesCS4B02G117900* and were located from 74.3 to 136.7 Mbp of 4BS encoding the myosin‐2 heavy chain‐like protein, B3 domain‐containing protein, transducin/WD40 repeat‐like superfamily protein, ATP‐dependent protease La (LON) domain‐containing protein, elongation factor Ts, and the SRP54. Based on the location of these six genes and single‐nucleotide polymorphisms (SNPs)/indels analysis, 49–137 Mbp of 4BS were suggested as the most probable interval for the *qDSI.4B.1* QTL (Figure 7A) (Nouraei et al., 2022).

In this study, according to the proteomic analysis of the same pair of NILs for *qDSI.4B.1* QTL, five proteins that were identified as proteins responsible for drought tolerance and one protein among the drought‐responsive proteins specific to the drought‐tolerant isoline had encoding genes located on 4BS. These six genes, including *TraesCS4B02G020300*, *TraesCS4B02G047700*, *TraesCS4B02G069300*, *TraesCS4B02G070300*, *TraesCS4B02G117900*, and *TraesCS4B02G124900*, were located in an interval from 14.4 to 151.5 Mbp of 4BS. Of these genes, *TraesCS4B02G069300* (63 Mbp), *TraesCS4B02G070300* (64.6 Mbp), and *TraesCS4B02G117900* (136.7 Mbp) that were located within the previously suggested interval of 49–137 Mbp, and *TraesCS4B02G047700* (35.5 Mbp) and *TraesCS4B02G124900* (151.5 Mbp) that were located within 15 Mbp upstream and downstream of this interval, were suggested as the candidate proteins responsible for drought tolerance in *qDSI.4B.1* QTL (Figure 7B and Table 4).

**TABLE 4:** Candidate proteins responsible for drought tolerance in the qDSI.4B.1 quantitative trait loci (QTL) located on the short arm of chromosome 4B (4BS).

Regulation^a^	Protein name^b^	Gene ID^c^	Physical position	Pathways^d^
Up	Uncharacterized protein	*TraesCS4B02G047700*	4B:35506264‐35515171	–
Down	Serine hydroxymethyltransferase	*TraesCS4B02G069300*	4B:62967286‐62972392	Glyoxylate and dicarboxylate metabolism
Down	Auxin‐repressed protein	*TraesCS4B02G070300*	4B:64611168‐64612597	–
Down	SRP54 domain‐containing protein	*TraesCS4B02G117900*	4B:136675381‐136680908	Protein export
Up	30S ribosomal protein S15	*TraesCS4B02G124900*	4B:151525915‐151526187	Ribosome

^a^ Up: higher expression in the tolerant isoline in the drought stress treatment; Down: higher expression in susceptible isoline in the drought stress treatment.

^b^ Protein name: recommended protein name according to the UniProt database (https://www.uniprot.org).

^c^ Gene ID: unique identifying number of the gene corresponding to the protein according to the IWGSC RefSeq v1.0 wheat assembly (https://wheat‐urgi.versailles.inra.fr).

^d^ Pathways: biological pathways in which the identified protein was found to be involved according to Kyoto Encyclopedia of Genes and Genomes (KEGG) database (https://www.kegg.jp/); *TraesCS4B02G117900* is the candidate gene in the current proteomic study that was also identified in the previous transcriptomics study (Nouraei et al., 2022).

The current study and relevant transcriptome study have confirmed the importance of *qDSI.4B.1* QTL in harboring candidate genes and/or proteins for drought tolerance. Although different source tissues (grain and leaves) were used for these two experiments, in both studies we found a common gene and its subsequent protein product (*TraesCS4B02G117900*, the SRP54) from this genomic region. Using a transgenic approach via gene editing and subsequent protein localization of this gene under DS is a suggested focus for further study.

### 4.4 Expression of the identified genes in various abiotic stresses

The expVIP analysis showed that the *TraesCS4B02G070300* gene had the highest expression among the five candidate genes for different abiotic stresses at the seedling and vegetative stages followed by *TraesCS4B02G069300*, which had high expression, especially in PEG 6000 osmotic stress, phosphorus starvation, and cold stress (Figure S15A). *TraesCS4B02G124900* had the highest expression in comparison to the other five genes in the spikes at the reproductive stage and the lowest expression as a result of phosphorus starvation at the vegetative stage. The exploration of the five candidate genes in WheatOmics showed that there was an expression of these genes in roots, leaves, and grains of a drought‐tolerant wheat genotype (Zubkov) under control and combined drought and heat stress conditions (Figure S15B). In the roots of Zubkov, *TraesCS4B02G069300* was downregulated, whereas *TraesCS4B02G117900*, *TraesCS4B02G047700*, *TraesCS4B02G124900*, and *TraesCS4B02G070300* were upregulated under stress condition, compared to control condition. A similar comparison in leaves indicated that the expression of all five genes was downregulated under combined drought and heat stress condition. This is in agreement with our finding that *TraesCS4B02G069300*, *TraesCS4B02G117900*, and *TraesCS4B02G124900* were downregulated in the TD_TC comparison (Table 3). In the grains, *TraesCS4B02G124900* was upregulated, whereas the rest of the genes were downregulated under stress compared to control condition (Figure S15B).

## 5 CONCLUSION

In this study, a comprehensive comparison of NILs for the *qDSI.4B.1* QTL with contrasting drought tolerance has been conducted based on morphological, physiological, and proteomic responses. The results have shown that the isoline with the positive allele from the tolerant parent C306 was comparatively more tolerant than the susceptible isoline with greater aerial parts, higher grain yield, and larger grains at maturity when water was withdrawn for 7 days at anthesis. From an iTRAQ‐based proteome analysis of the leaf samples collected at the end of the period of water stress and combining various pairwise comparisons between isolines, the proteins responsible for drought tolerance were identified. These proteins were mainly enriched in hydrogen peroxide metabolic activity, ROS metabolic activity, photosynthetic activity, intracellular protein transport, cellular protein localization, cellular macromolecule localization, and response to oxidative stress. Prediction of interactions and pathway analysis for tolerant conferring proteins revealed broad interaction between proteins in assigned pathways of transcription, translation, protein export, photosynthesis, and carbohydrate metabolism. Overall, the five proteins with their encoding genes located on 4BS were suggested as the proteins responsible for drought tolerance in the *qDSI.4B.1* QTL with one (*TraesCS4B02G117900*) reported for conferring drought tolerance in our previous transcriptomic study. The results of this study provide more insights into the molecular mechanisms underpinning drought tolerance in wheat.

## AUTHOR CONTRIBUTIONS

**Sina Nouraei**: Conceptualization; data curation; formal analysis; investigation; methodology; validation; visualization; writing–original draft. **Md Sultan Mia**: Conceptualization; methodology; resources; supervision; writing–review and editing. **Hui Liu**: Conceptualization; funding acquisition; methodology; resources; supervision; writing–review and editing. **Neil C. Turner**: Conceptualization; methodology; supervision; writing–review and editing. **Javed M. Khan**: Data curation; methodology; writing–review and editing. **Guijun Yan**: Conceptualization; funding acquisition; methodology; project administration; resources; supervision; writing–review and editing.

## CONFLICT OF INTEREST STATEMENT

The authors declare that they have no known conflict of interests or personal relationships that could influence the work reported in this paper.

## Supporting information

Supplementary figures S1‐S15

**Table S1** Drought tolerance inducing differentially expressed proteins found between the tolerant and susceptible isolines (TD_SD).

**Table S2** Drought responsive differentially expressed proteins specifically found in the tolerant isoline (TD_TC).

**Table S3** Drought responsive differentially expressed proteins specifically found in the susceptible isoline (SD_SC).

**Table S4** Summary of genes and gene‐specific primers used for qRT‐PCR.
