# Supplementary material for: Proteomic analysis of near‐isogenic lines reveals key biomarkers on wheat chromosome 4B conferring drought tolerance
Source: Plant Genome. 2023 May 18;17(1):e20343. doi: 10.1002/tpg2.20343 (PMC12807022; doi:10.1002/tpg2.20343)
Supplement: Supplementary file 1 [file TPG2-17-e20343-s003.pdf]

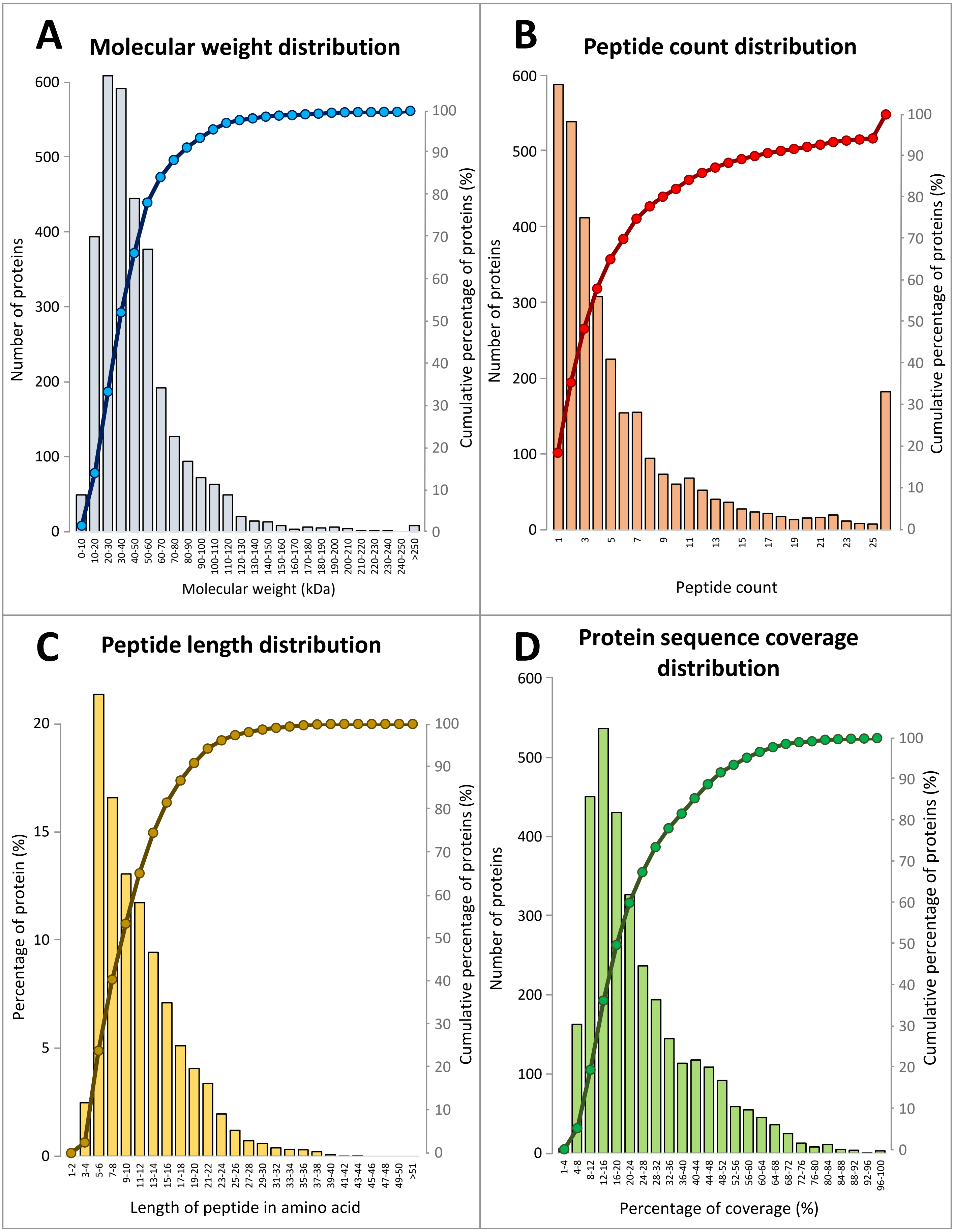

Supplementary Figure S1: General information of iTRAQ data output. (A) molecular weight, (B) peptide count, (C) peptide length, and (D) protein sequence coverage distribution for the identified proteins.

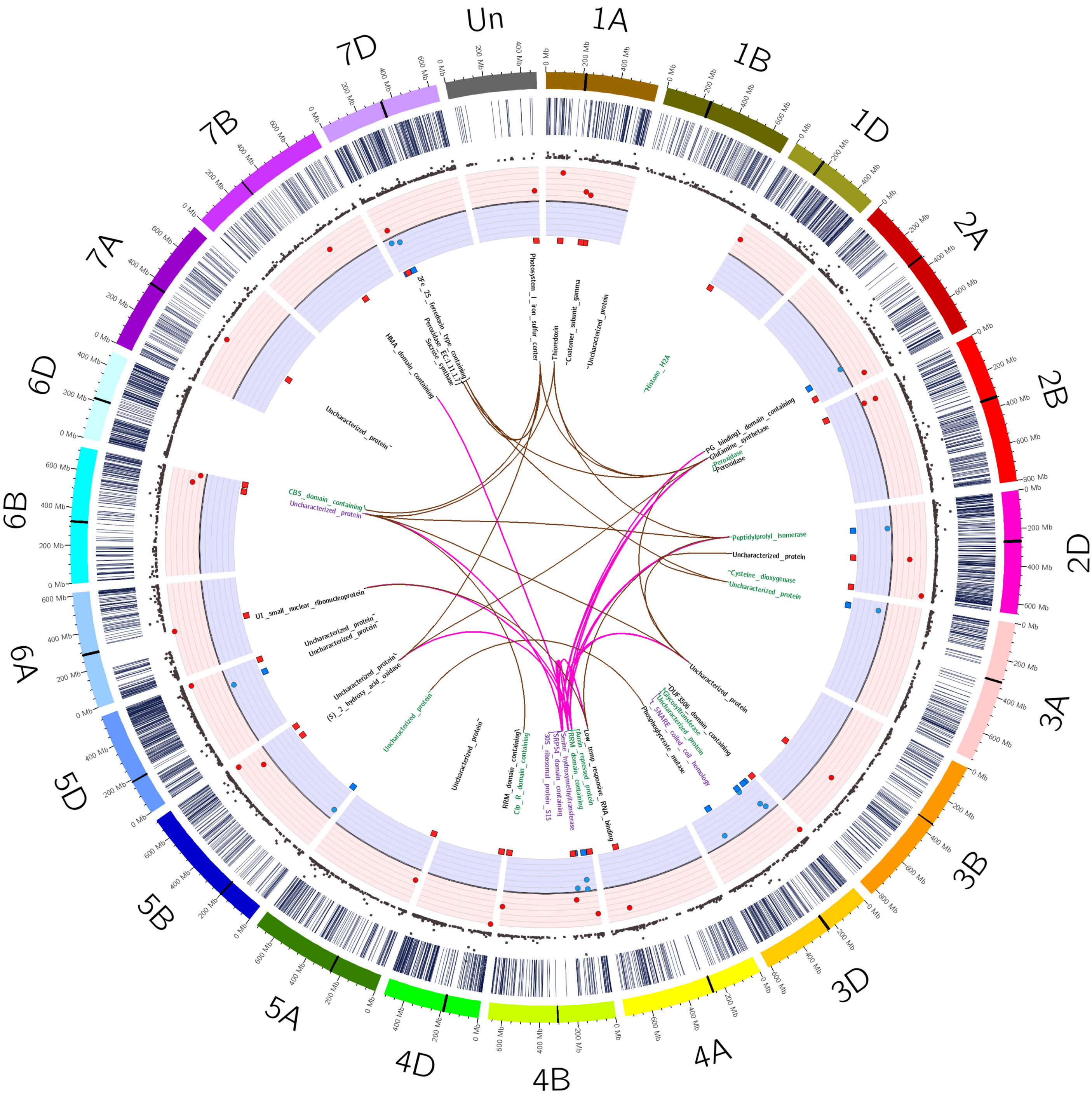

Supplementary Figure S2: Circos plot showing the distribution of 41 proteins responsible for drought tolerance mapped to different chromosomes of wheat.

Circles: (1) chromosomes (thick lines are centromeres); (2) all identified proteins; (3) peptide count for all identified proteins; (4) scatter plot showing log2 of foldchanges for 41 identified proteins responsible for drought tolerance in coloured circles, red colour represents up-regulation and blue represents down-regulation; (5) red and blue squares representing regulation and location of 41 proteins; (6) text plot showing name of proteins, green text represents 12 proteins specific to TD_SD comparison (Area I-a) and purple text represents 5 proteins shared between TD_SD and TD_TC (Area I-b); (7) link tracks illustrates the interaction between proteins according to protein-protein interaction analysis through the STRING database (https://string-db.org/), pink links represent interactions of proteins located on 4BS with other proteins. Un: unknown chromosome including mitochondria and chloroplast proteins.

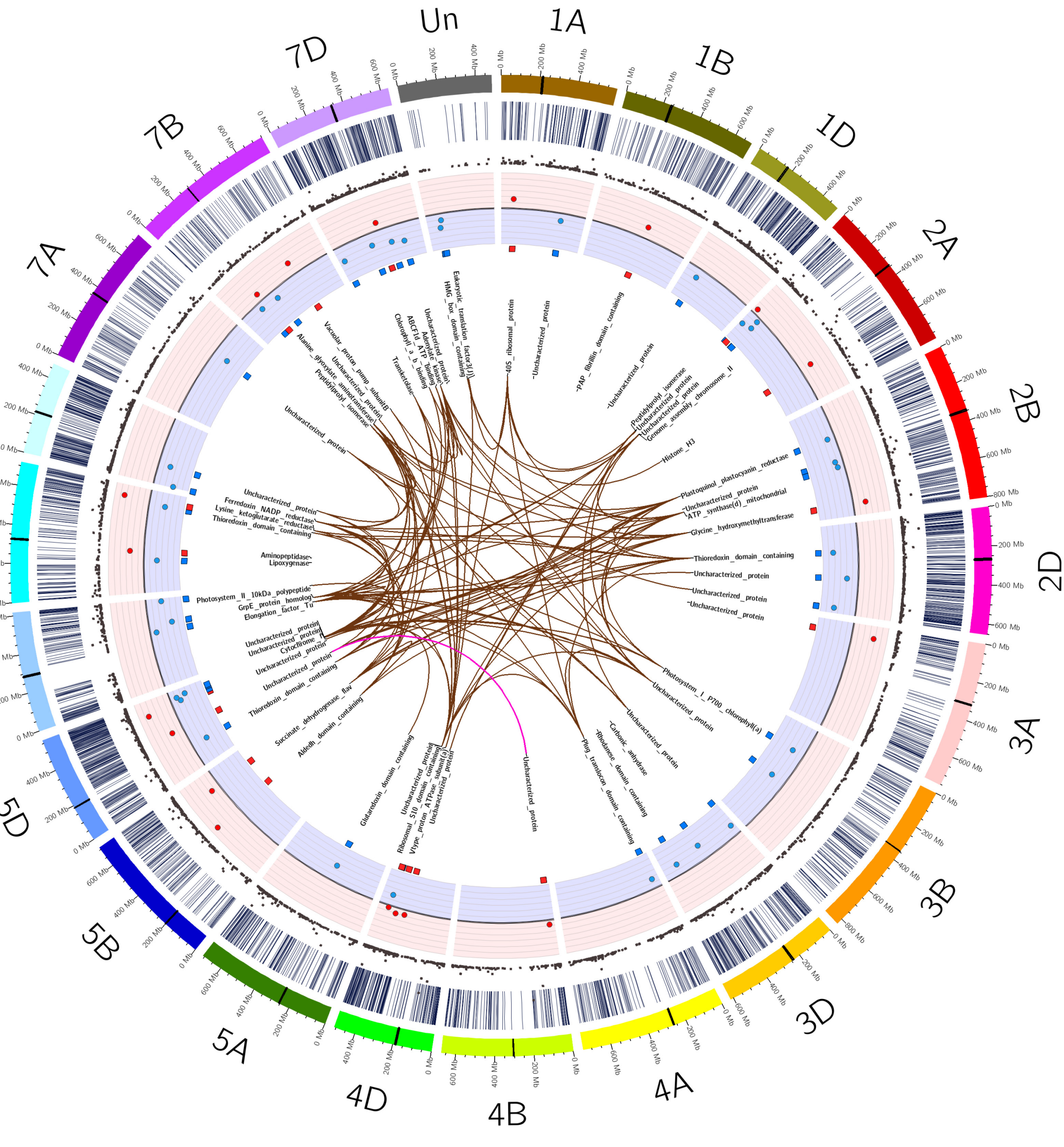

Supplementary Figure S3: Circos plot showing the distribution of 58 drought responsive proteins specific to the drought tolerant isoline mapped to different chromosomes of wheat.

Circles: (1) chromosomes (thick lines are centromeres); (2) all identified proteins; (3) peptide count for all identified proteins; (4) scatter plot showing log2 of foldchanges for 58 drought responsive proteins specific to the drought tolerant isoline in coloured circles, red colour represents up-regulation and blue represents down-regulation; (5) red and blue squares representing regulation and location of 58 proteins; (6) text plot showing name of proteins; (7) link tracks illustrating interaction between proteins according to protein-protein interaction analysis through the STRING database (https://string-db.org/), pink link represents interaction of protein located on 4BS with another protein. Un: unknown chromosome including mitochondria and chloroplast proteins.

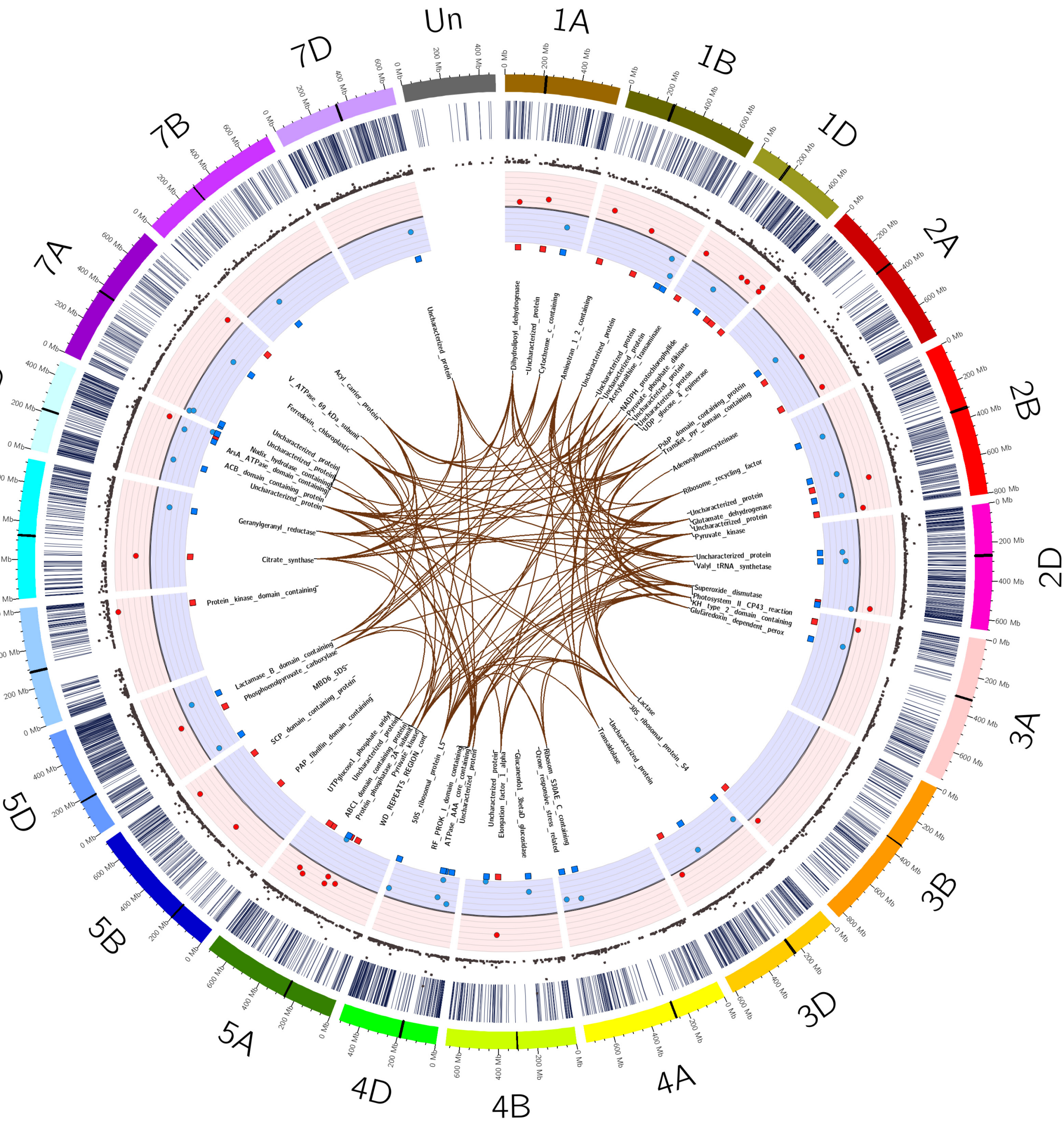

Supplementary Figure S4: Circos plot showing the distribution of 64 drought responsive proteins specific to the drought susceptible isoline mapped to different chromosomes of wheat.

Circles: (1) chromosomes (thick lines are centromeres); (2) all identified proteins; (3) peptide count for all identified proteins; (4) scatter plot showing log2 of foldchanges for 64 drought responsive proteins specific to the drought susceptible isoline in coloured circles, red colour represents up-regulation and blue represents down-regulation; (5) red and blue squares representing regulation and location of 64 proteins; (6) text plot showing name of proteins; (7) link tracks illustrating interaction between proteins according to protein-protein interaction analysis through the STRING database (https://string-db.org/). Un: unknown chromosome including mitochondria and chloroplast proteins.

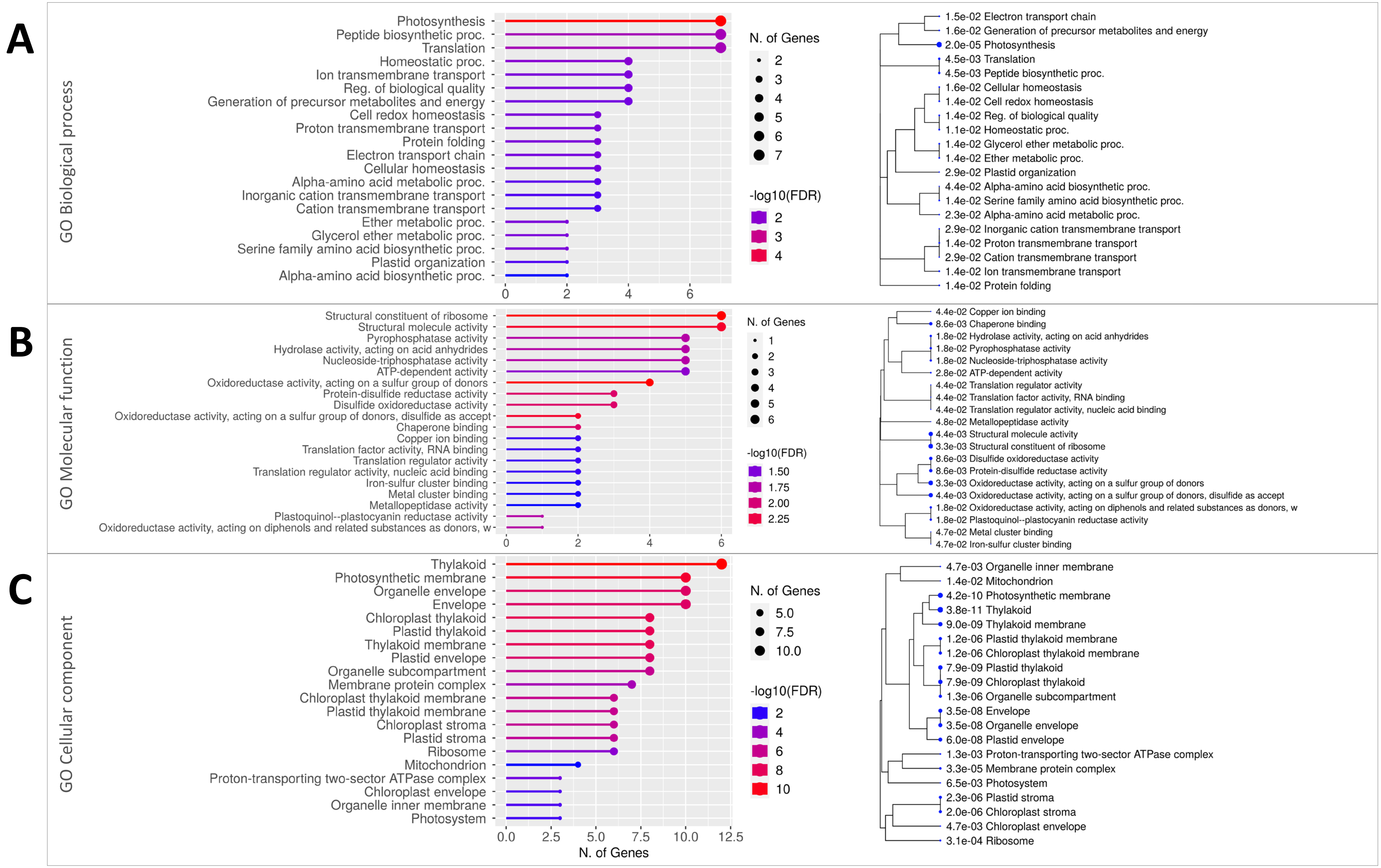

Supplementary Figure S5: Gene ontology (GO) enrichment analysis of drought responsive proteins specific to the drought tolerant isoline. Lollipop charts on left show the top enriched pathways in (A) biological process (B) molecular function, and (C) cellular component with FDR < 0.05. X-axis represents the number of genes (proteins); Y-axis displays the functional categories. Hierarchical clustering trees on right summarize the correlation among significant enriched pathways listed on the left. Pathways with many shared genes are clustered together and bigger dots indicate more significant *p*-values.

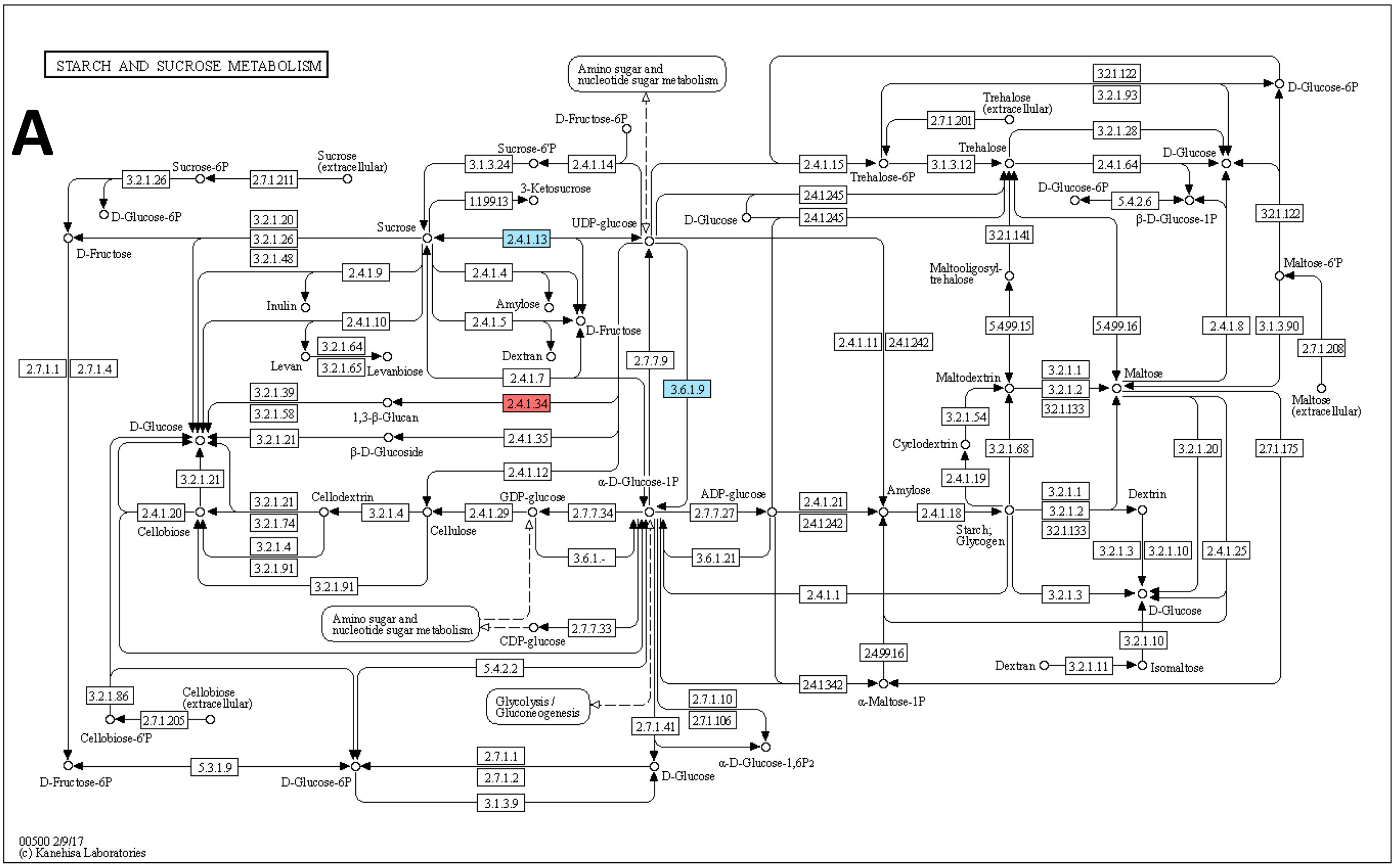

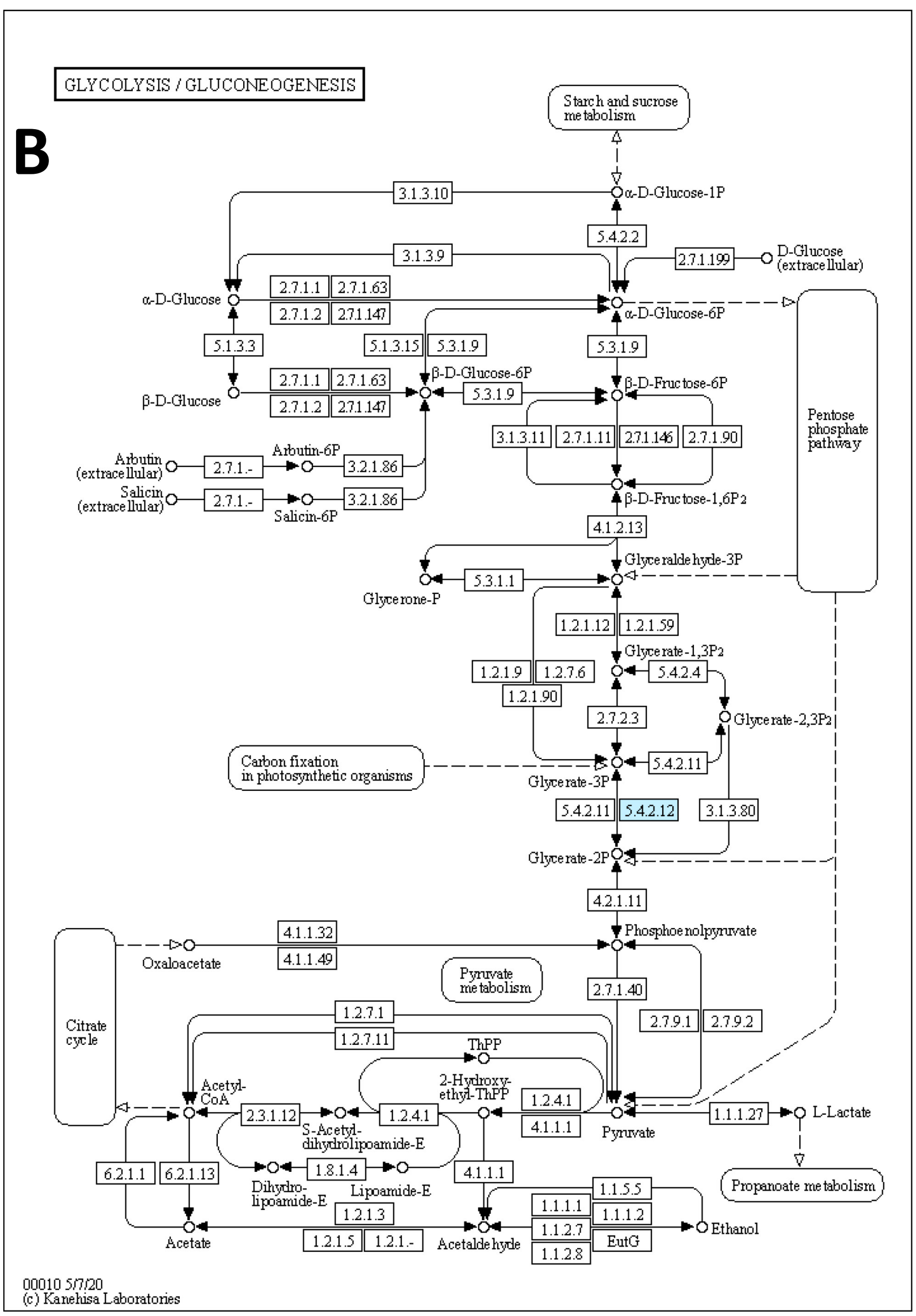

Supplementary Figure S6: (A) Starch and sucrose metabolism, and (B) glycolysis/gluconeogenesis pathway derived from Kyoto Encyclopedia of Genes and Genomes (KEGG) (https://www.kegg.jp/). Proteins in red or blue boxes were found as proteins responsible for drought tolerance in TD_SD comparison; colour intensity corresponding to the foldchange values; proteins in red had higher and in blue had lower abundance in the tolerant compared to the susceptible isoline under drought stress; [EC:2.4.1.13]: sucrose synthase; [EC:3.6.1.9]: ectonucleotide pyrophosphatase/phosphodiesterase family member 1/3; [EC:2.4.1.34]: 1,3-beta-glucan synthase; [EC:5.4.2.12]: 2,3-bisphosphoglycerate-independent phosphoglycerate mutase.

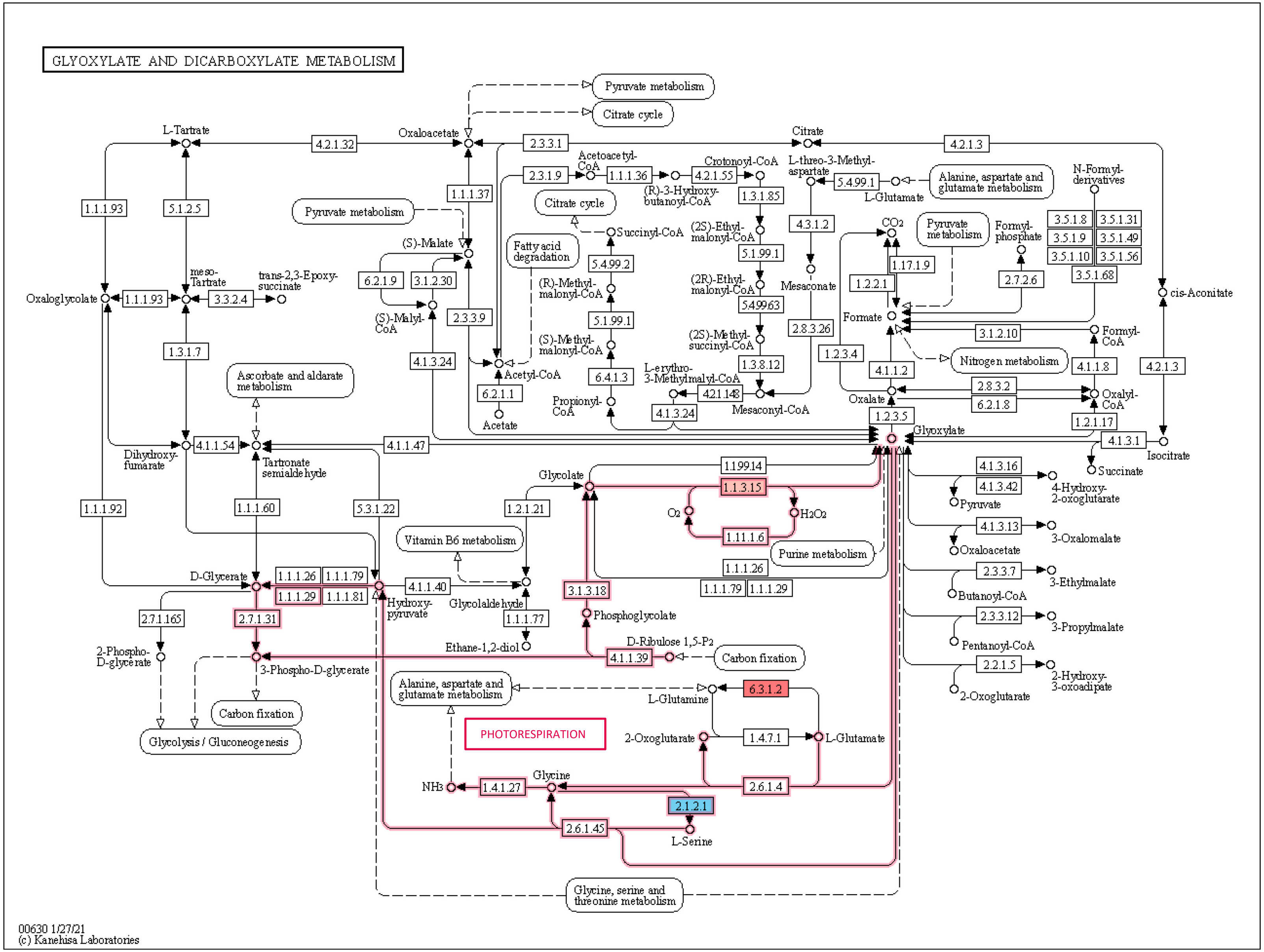

Supplementary Figure S7: The glyoxylate and dicarboxylate metabolism derived from the Kyoto Encyclopedia of Genes and Genomes (KEGG) (https://www.kegg.jp/). Proteins in red or blue boxes were found as proteins responsible for drought tolerance in the TD_SD comparison; colour intensity corresponding to the foldchange values; proteins in red had higher and in blue had lower abundance in the tolerant compared to the susceptible isoline under drought stress; [EC:1.1.3.15]: (S)-2-hydroxy-acid oxidase; [EC:6.3.1.2]: glutamine synthetase; [EC:2.1.2.1]: serine hydroxymethyltransferase.

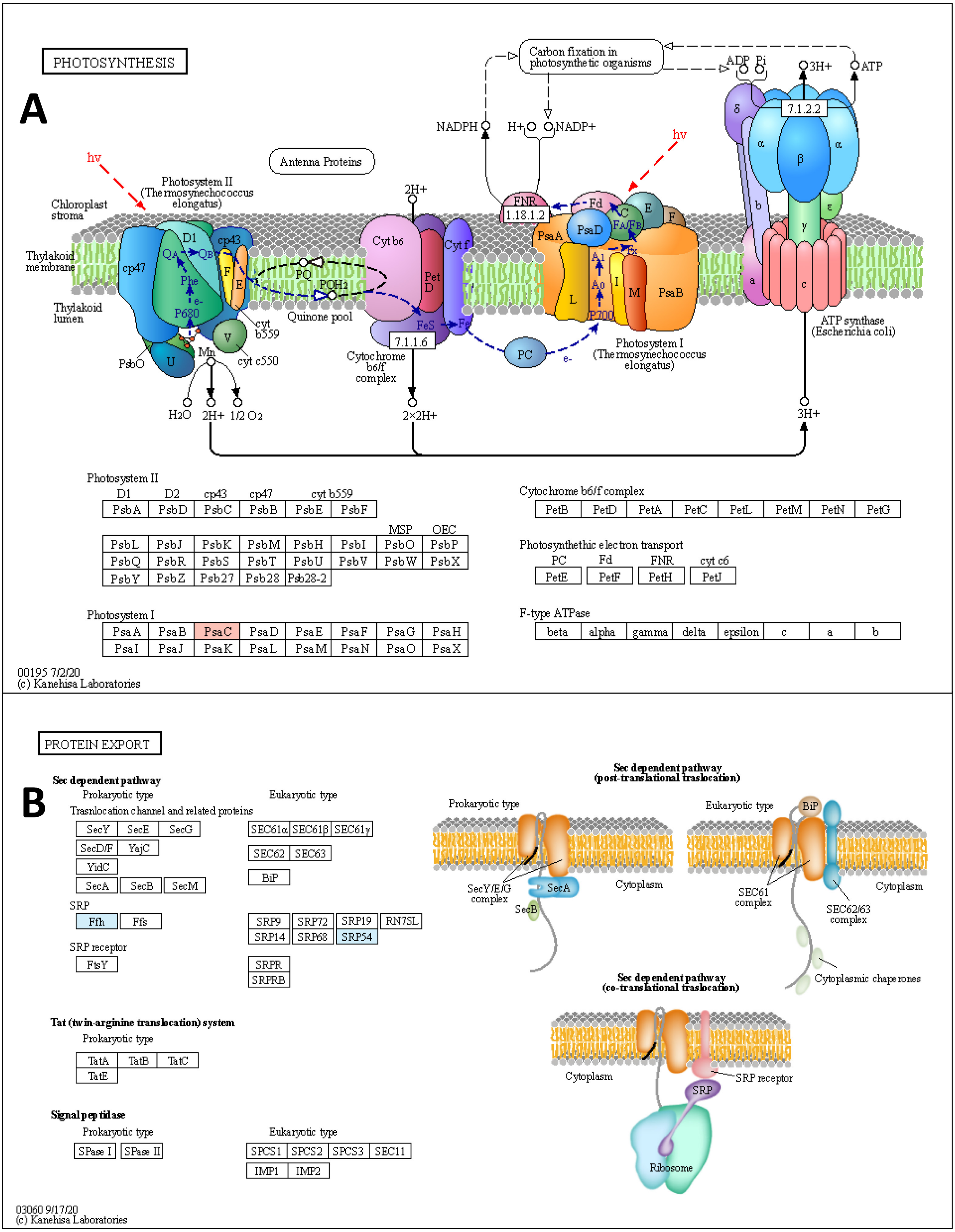

Supplementary Figure S8: (A) photosynthesis, and (B) protein export pathways derived from the Kyoto Encyclopedia of Genes and Genomes (KEGG) (https://www.kegg.jp/). Proteins in red or blue boxes were found as proteins responsible for drought tolerance in the TD_SD comparison; colour intensity corresponds to the foldchange values; proteins in red had higher and in blue had lower abundance in the tolerant compared to the susceptible isoline under drought stress; [PsaC]: photosystem I iron-sulfur subunit; [SRP54]: [Ffh]: signal recognition particle subunit SRP54.

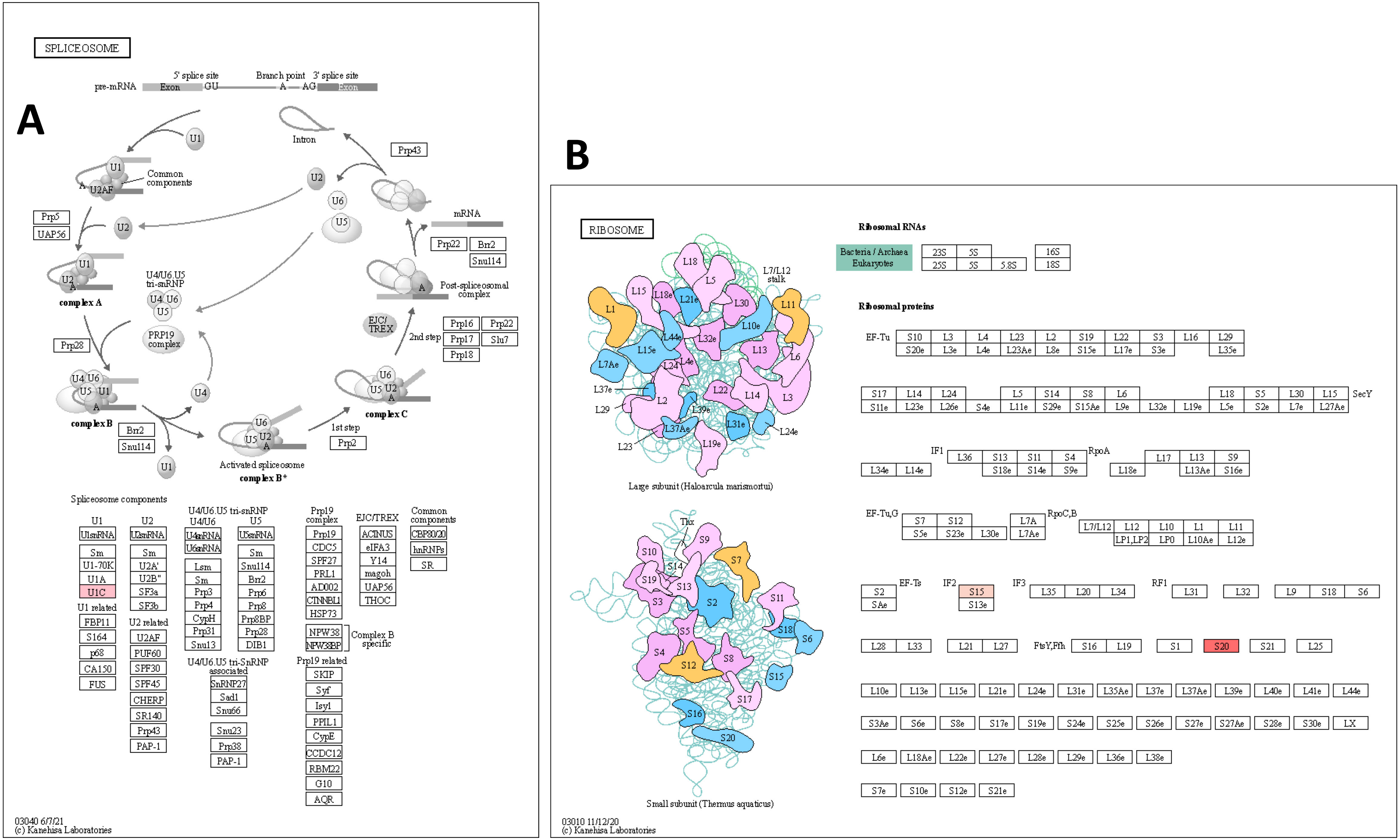

Supplementary Figure S9: (A) Spliceosome, and (B) ribosome complex derived from the Kyoto Encyclopedia of Genes and Genomes (KEGG) (https://www.kegg.jp/). Proteins in red or blue boxes were found as proteins responsible for drought tolerance in the TD_SD comparison; colour intensity corresponds to the foldchange values; proteins in red had higher and in blue had lower abundance in the tolerant compared to the susceptible isoline under drought stress; [U1C]: U1 small nuclear ribonucleoprotein C; [S15]: 30S ribosomal protein S15; [S20]: small subunit ribosomal protein S20.

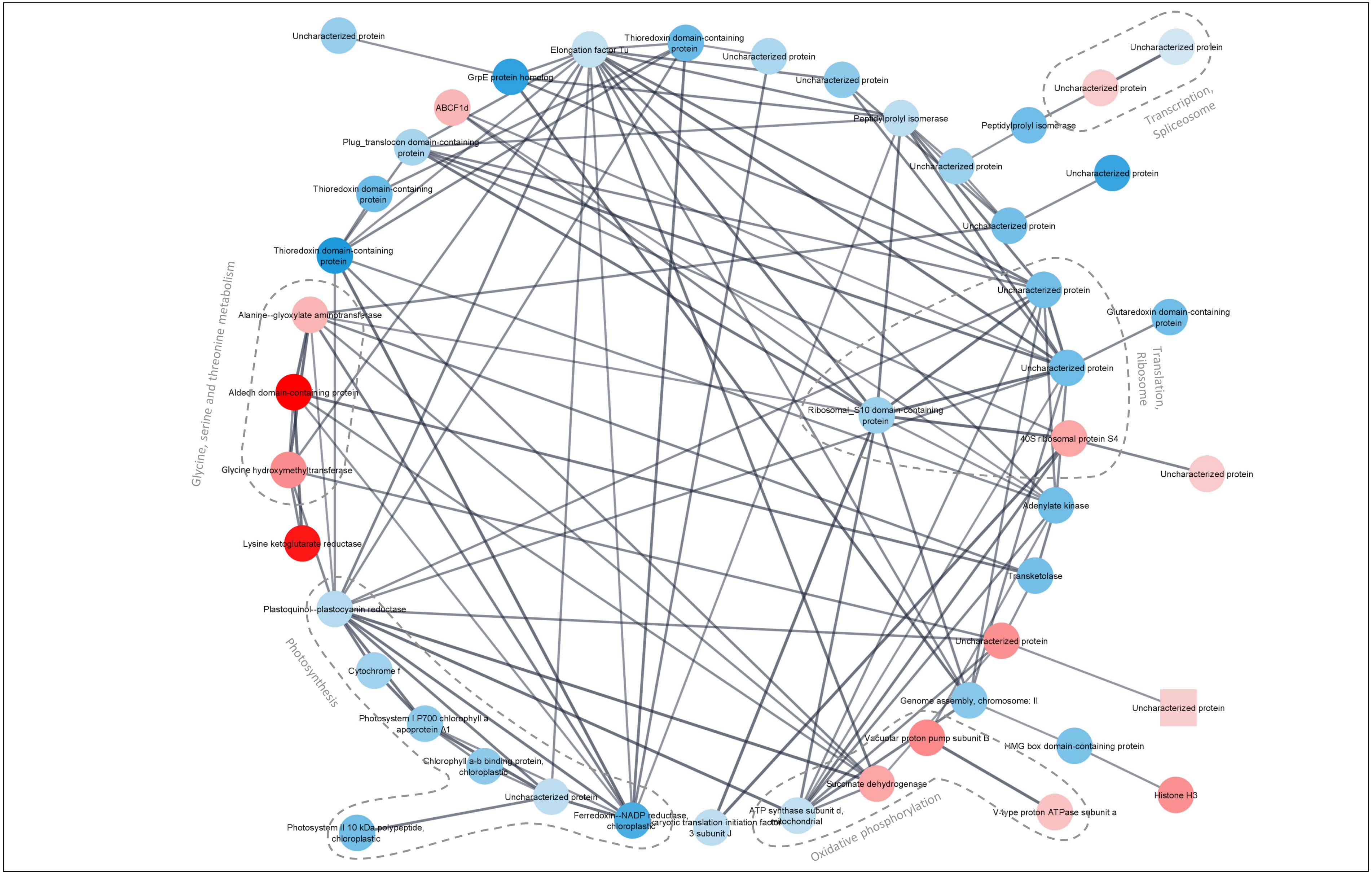

Supplementary Figure S10: Protein interaction network of drought responsive proteins specific to the drought tolerant isoline. The network is predicted through the STRING database (https://string-db.org/) and Cytoscape v3.9.1 (https://cytoscape.org/) with confidence score higher than 0.5. Nodes representing proteins; colour intensity corresponds to the foldchange values; red proteins were up-regulated and blue proteins were down-regulated after stress in the tolerant isoline; line thickness represents the strength of the supporting data. Dotted lines show pathway groups base on the Kyoto Encyclopedia of Genes and Genomes (KEGG) pathway analysis (https://www.kegg.jp/). Protein with encoding gene located on 4BS is shown in square.

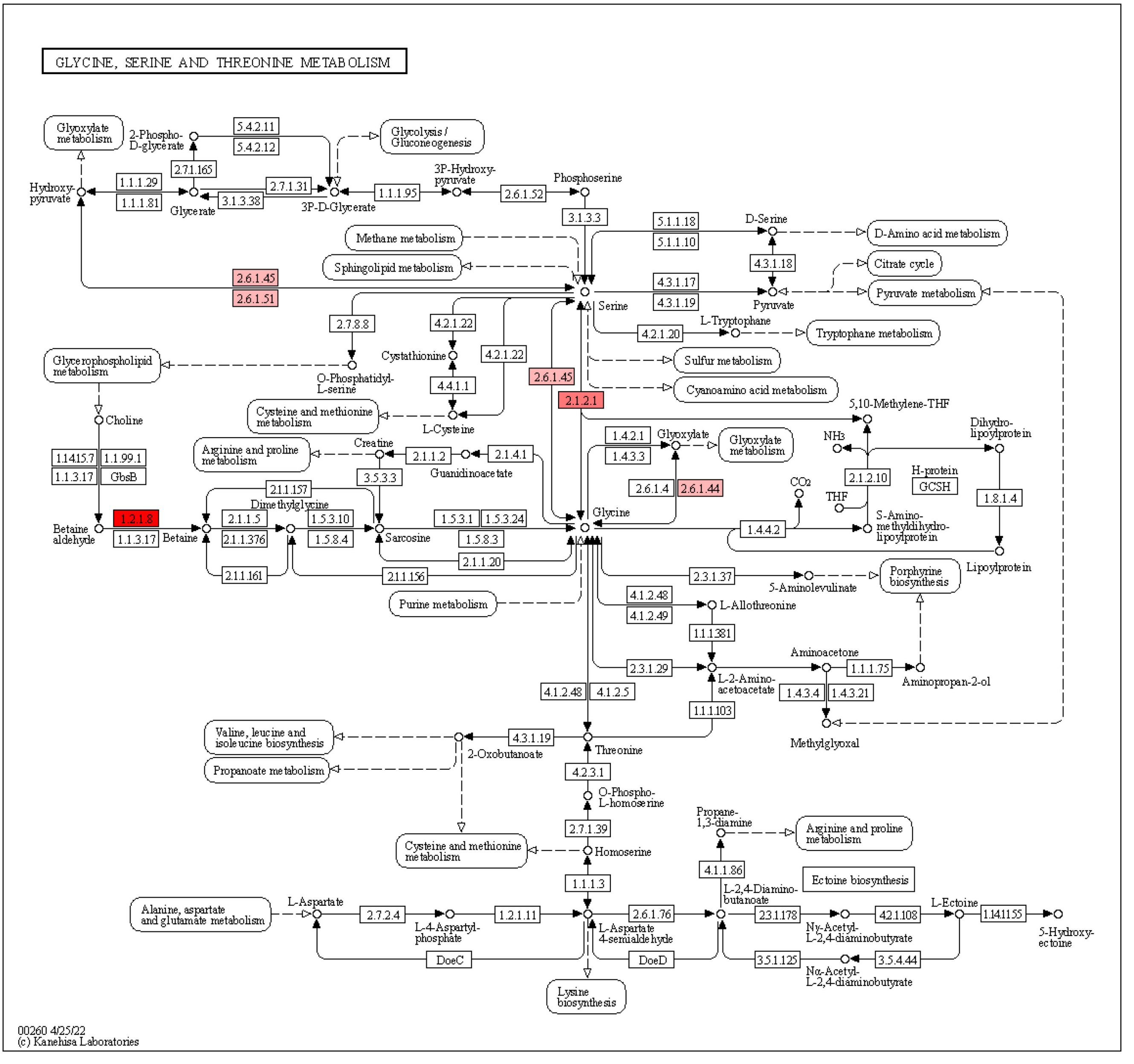

Supplementary Figure S11: Glycine, serine and threonine metabolism derived from the Kyoto Encyclopedia of Genes and Genomes (KEGG) (https://www.kegg.jp/). Proteins in red or blue boxes were found as drought responsive proteins specific to the drought tolerant isoline in the TD_TC comparison; colour intensity corresponds to the foldchange values; red proteins were up-regulated and blue proteins were down-regulated after stress in the tolerant isoline; [EC:2.6.1.44 2.6.1.45 2.6.1.51]: alanine-glyoxylate transaminase / serine-glyoxylate transaminase / serine-pyruvate transaminase; [EC:2.1.2.1]: glycine hydroxymethyltransferase; [EC:1.2.1.8]: Aldedh domain-containing protein.

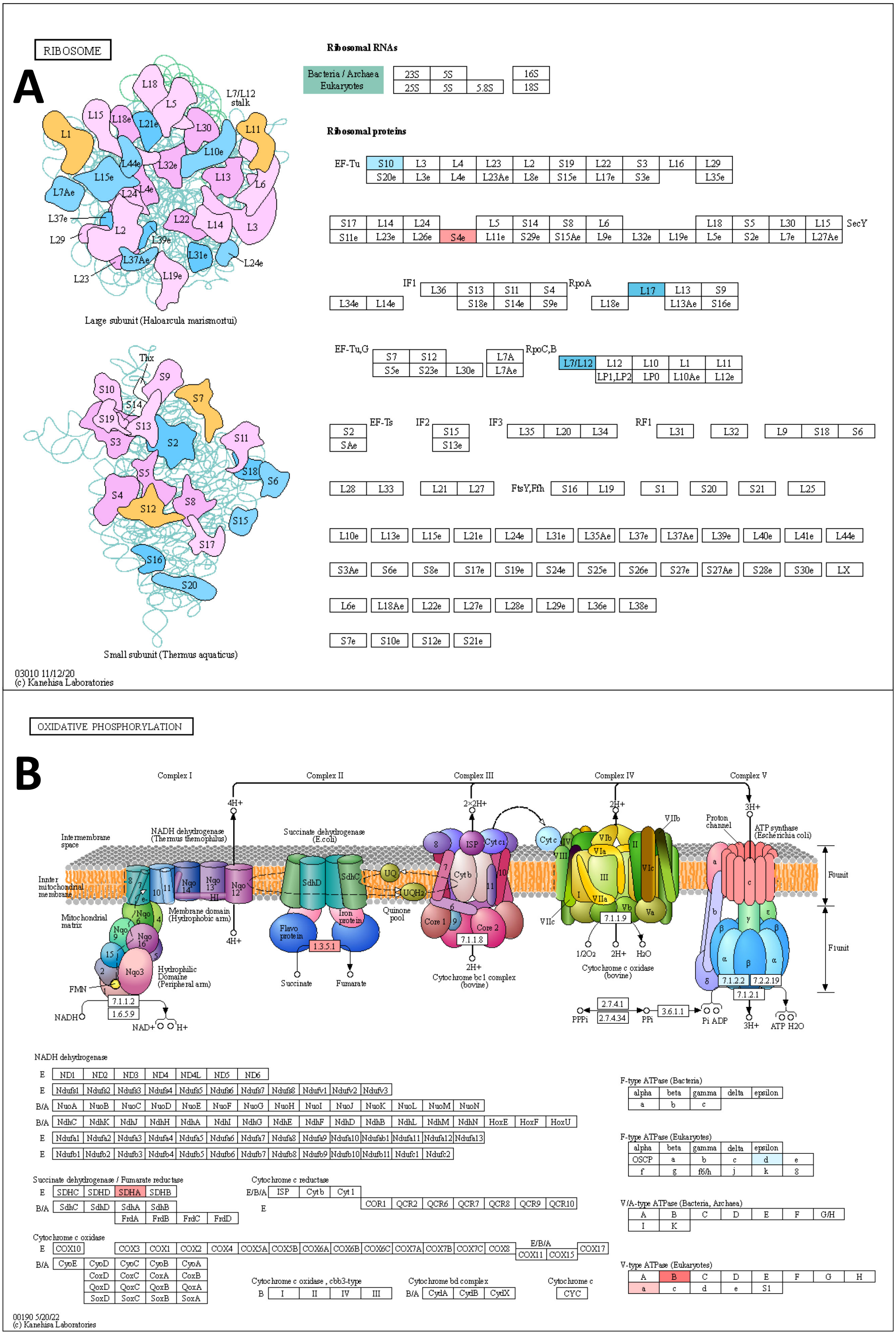

Supplementary Figure S12: (A) Ribosome, and (B) oxidative phosphorylation pathways derived from the Kyoto Encyclopedia of Genes and Genomes (KEGG) (https://www.kegg.jp/). Proteins in red or blue boxes were found as drought responsive proteins specific to the drought tolerant isoline in the TD_TC comparison; colour intensity corresponds to the foldchange values; red proteins were up-regulated and blue proteins were down-regulated after stress in tolerant isoline; [S10]: small subunit ribosomal protein S10; [S4e]: 40S ribosomal protein S4e; [L17]: large subunit ribosomal protein L17; [L7/L12]: large subunit ribosomal protein L7/L12; [EC:1.3.5.1]: [SDHA]: succinate dehydrogenase; [d]: ATP synthase subunit d; [a]: V-type proton ATPase subunit a; [B]: vacuolar proton pump subunit B.

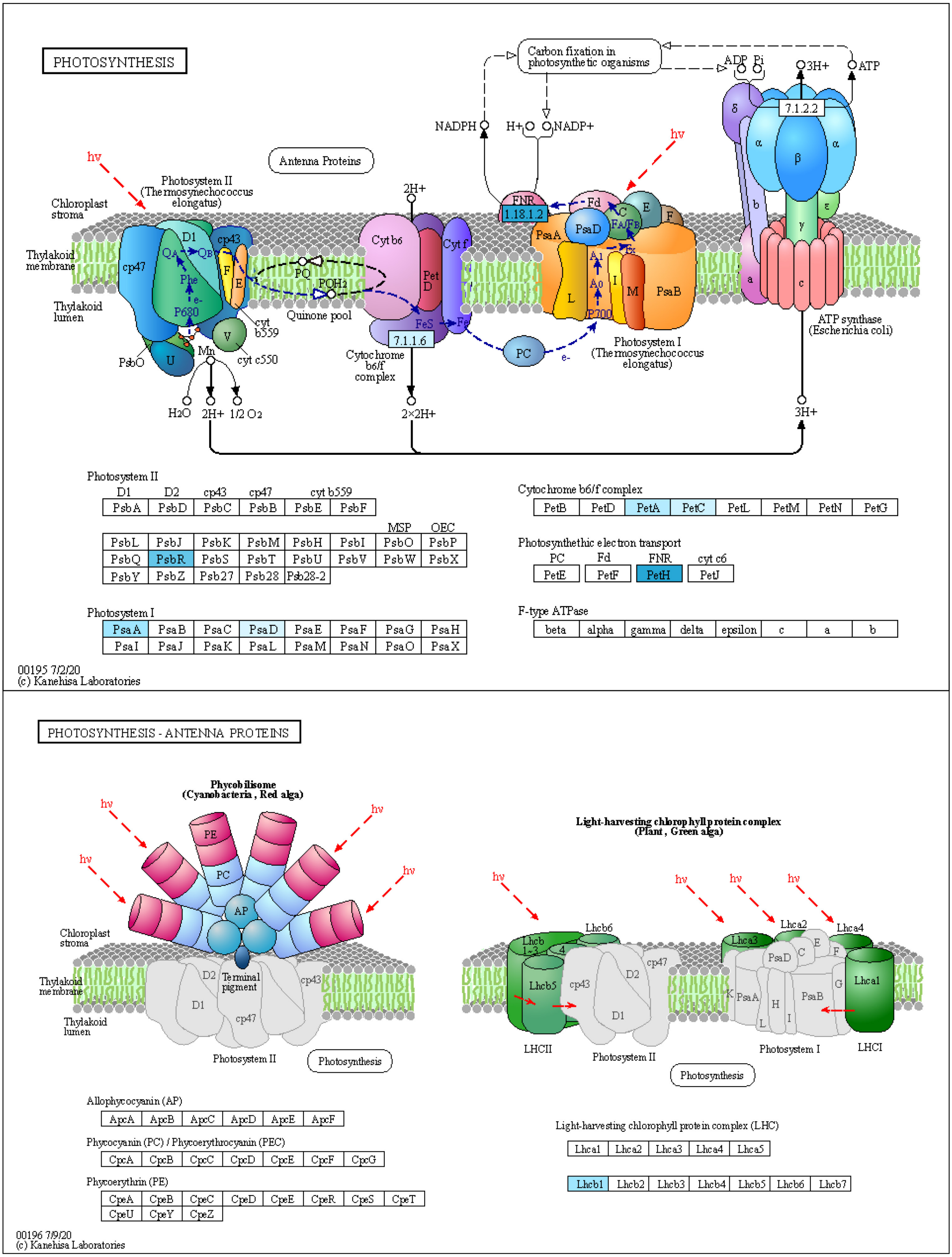

Supplementary Figure S13: (A) Photosynthesis, and (B) photosynthesis – antenna proteins derived from the Kyoto Encyclopedia of Genes and Genomes (KEGG) (https://www.kegg.jp/). Proteins in red or blue boxes were found as drought responsive proteins specific to the drought tolerant isoline in the TD_TC comparison; colour intensity corresponds to the foldchange values; red proteins were up-regulated and blue proteins were down-regulated after stress in the tolerant isoline; [petA]: cytochrome f; [EC:7.1.1.6]: [petC]: plastoquinol--plastocyanin reductase; [psbR]: photosystem II 10 kDa polypeptide; [EC:1.18.1.2]: [petH]: ferredoxin--NADP reductase; [psaA]: photosystem I P700 chlorophyll a apoprotein A1; [psaD]: photosystem I subunit II; [Lhcb1]: chlorophyll a-b binding protein.

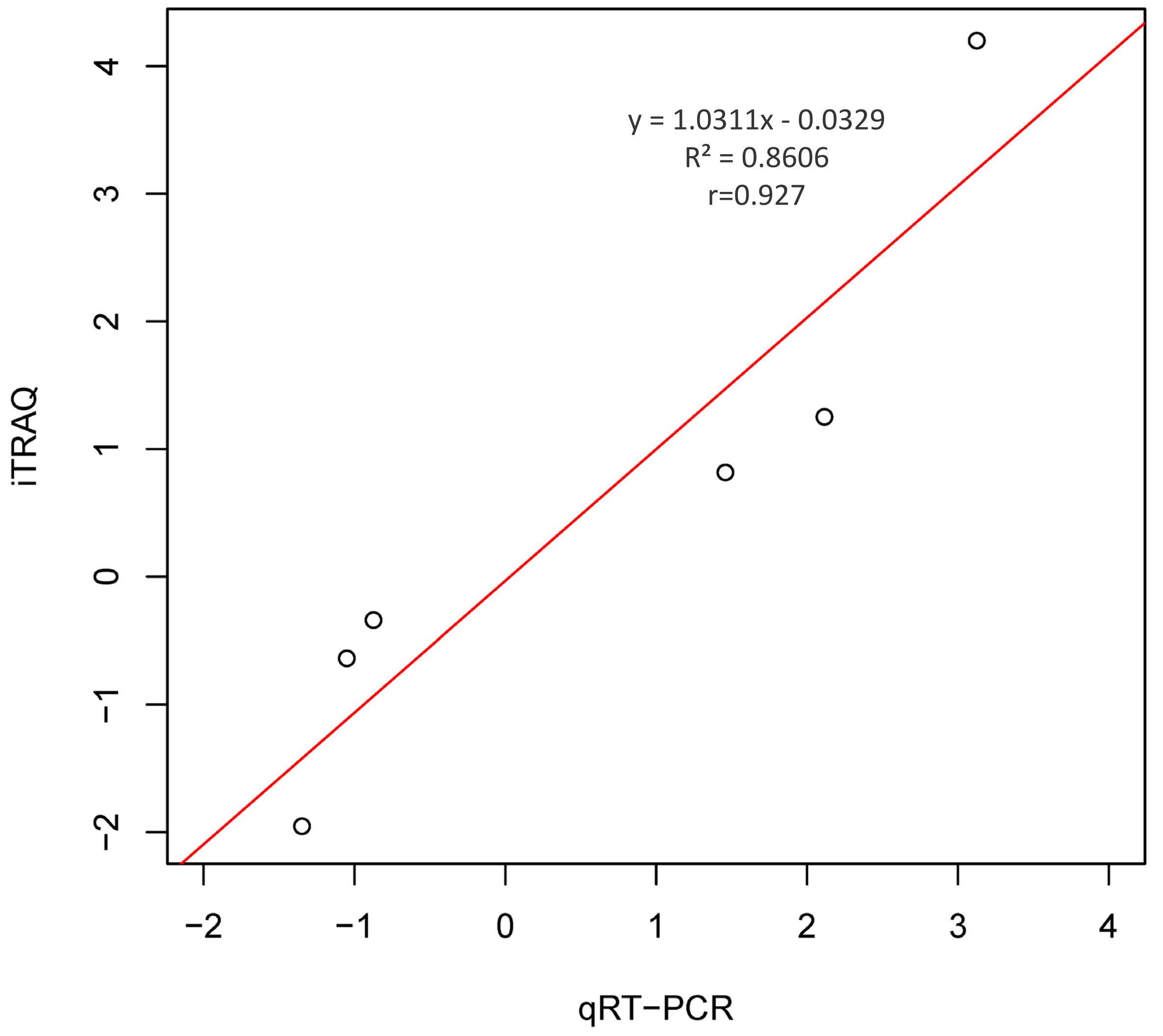

Supplementary Figure S14: Scatter plot and regression line of iTRAQ versus qRT-PCR measurements for six candidate genes responsible for drought tolerance on short arm of chromosome 4B. X-axis indicates log2 of qRT-PCR fold changes and Y-axis indicates log2 of iTRAQ fold changes. In the linear equation of $y = mX + b$, m is the slope of the line and b is the intercept. R^2 is R-square showing qRT-PCR can explain 86% of the variation in iTRAQ data.

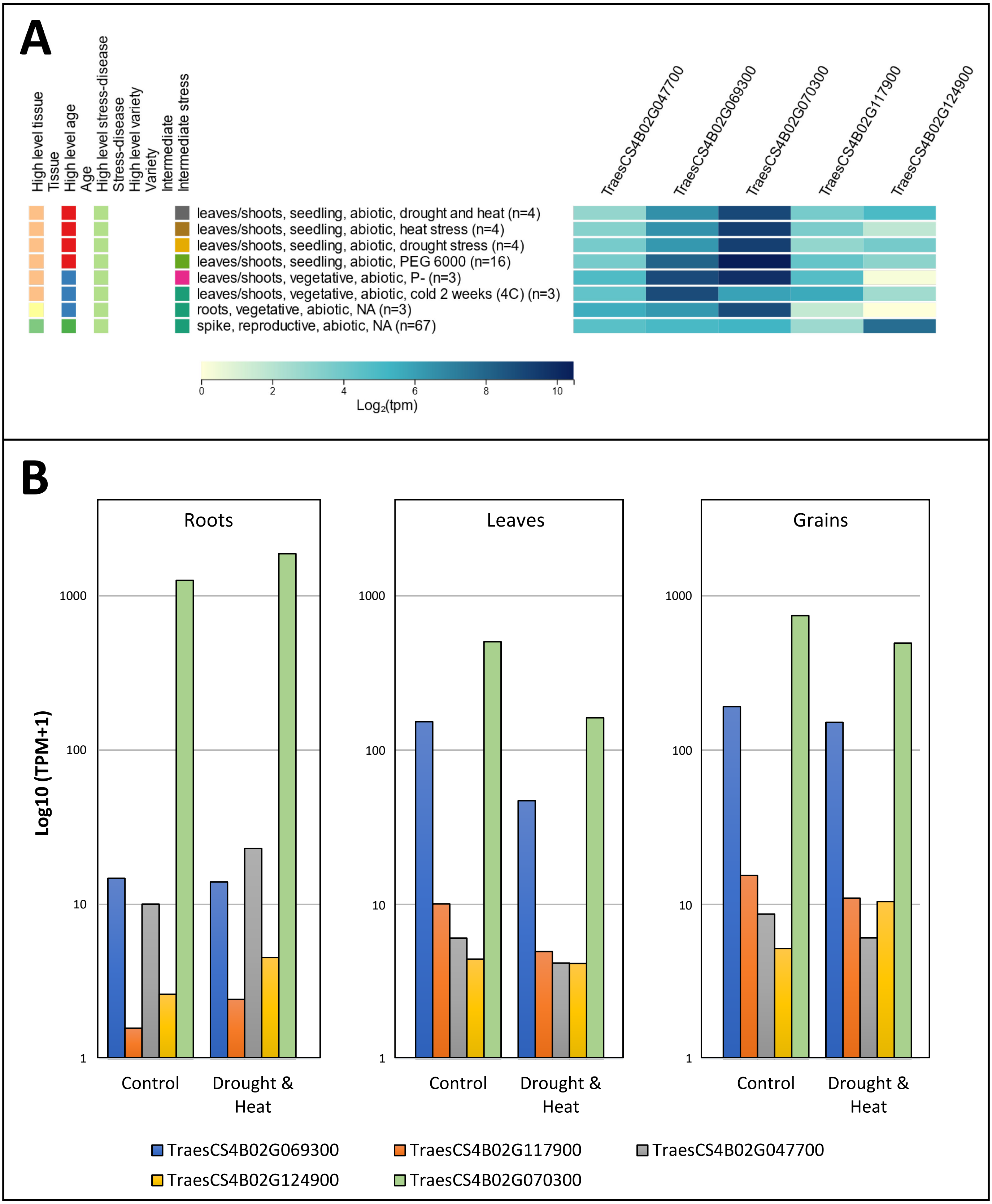

Supplementary figure S15: (A) Heatmap showing the expression of the five candidate genes under different abiotic stresses based on the analysis in the public transcriptomic database of wheat (expVIP, http://www.wheat-expression.com), (B) expression of the five candidate genes in a drought tolerant genotype (Zubkov) under control and combined drought and heat stress conditions based on the data from the wheat multi-omics database (WheatOmics, http://202.194.139.32/expression/wheat.html). "n" is the number of samples that were averaged to produce the expression values, "TPM" is transcript per million, "P-" is phosphorus starvation, and "NA" is not available.
